# Vasopressin receptor 2 mutations in the nephrogenic syndrome of inappropriate antidiuresis show different mechanisms of constitutive activation for G protein coupled receptors

**DOI:** 10.1038/s41598-020-65996-w

**Published:** 2020-06-04

**Authors:** Vanessa Vezzi, Caterina Ambrosio, Maria Cristina Grò, Paola Molinari, Gökçe Süral, Tommaso Costa, H. Ongun Onaran, Susanna Cotecchia

**Affiliations:** 1 Istituto Superiore di Sanitá, National Center for Drug Research and Evaluation, Rome, Italy; 2 Ankara University, Faculty of Medicine, Department of Pharmacology, Molecular biology and Technology development unit, Sıhhiye, Ankara, Turkey; 3 Department of Biosciences, Biotechnologies and Biopharmaceutics, University of Bari, 70125 Bari, Italy

**Keywords:** G protein-coupled receptors, Cell signalling, Receptor pharmacology

## Abstract

Vasopressin receptor 2 (V2R) mutations causing the nephrogenic syndrome of inappropriate antidiuresis (NSIAD) can generate two constitutively active receptor phenotypes. One type results from residue substitutions in several V2R domains and is sensitive to vaptan inverse agonists. The other is only caused by Arg 137 replacements and is vaptan resistant. We compared constitutive and agonist-driven interactions of the vaptan-sensitive F229V and vaptan-resistant R137C/L V2R mutations with β-arrestin 1, β-arrestin 2, and Gαs, using null fibroblasts reconstituted with individual versions of the ablated transduction protein genes. F229V displayed very high level of constitutive activation for Gs but not for β-arrestins, and enhanced or normal responsiveness to agonists and inverse agonists. In contrast, R137C/L mutants exhibited maximal levels of constitutive activation for βarrestin 2 and Gs, minimal levels for β-arrestin 1, but a sharp decline of ligands sensitivity at all transducer interactions. The enhanced constitutive activity and reduced ligand sensitivity of R137 mutants on cAMP signaling persisted in cells lacking β-arrestins, indicating that these are intrinsic molecular properties of the mutations, not the consequence of altered receptor trafficking. The results suggest that the two groups of NSIAD mutations represent two distinct molecular mechanisms of constitutive activation in GPCRs.

## Introduction

Spontaneous signaling of the vasopressin 2 receptor (V2R) is responsible for the Nephrogenic Syndrome of Inappropriate Antidiuresis (NSIAD), a rare genetic disease characterized by decreased renal water excretion, hyponatremia and undetectable vasopressin circulating levels^1–3^.

The first mutations of NSIAD were characterized in two male infants with hyponatremia and involved the substitution of Arg 137 with either Cys or Leu (R137C or R137L) at the cytosolic end of helix 3 in V2R^1^. This GPCR-wide conserved Arg^4^ is part of the well-known consensus motif E/DRY/H and plays a crucial role in the mechanism of receptor activation, as indicated by structural data^5,6^.

Another natural substitution of codon 137 identified earlier in V2R studies is R137H^7^, but unlike NSIAD mutations, it generates a loss-of-function phenotype^8^ that occurs in patients with the X-linked form of congenital Nephrogenic Diabetes Insipidus (NDI). This is a genetic disorder characterized by a failure to concentrate urine under normal or elevated levels of arginine vasopressin (AVP), polyuria, polydipsia and hypernatremia^9^. Thus, mutations in position 137 can result in loss or gain of receptor function, depending on the residue replacing Arg.

However, activating NSIAD mutations were also discovered in different V2R regions, including, F229V at the cytosolic end of transmembrane helix 5^10^, I130N in helix 3^11^, and L312S in helix 7^12^. Based on biological and pharmacological traits, NSIAD mutants can be divided in two different functional groups.

One group consists of codon 137 mutants, which are characterized by decreased membrane expression, weak AVP stimulation, constitutive β−arrestin 2 recruitment, and a moderate level of constitutive activation for cAMP signaling. Most importantly, the constitutive activity of these receptors cannot be inhibited by vaptan inverse agonists^13–15^.

The other group includes F229V, I130N and L312S mutants, displaying normal membrane expression and AVP response, lack of constitutive interaction with β−arrestin 2, but strong and vaptan-sensitive constitutive activation of cAMP signaling^10–12^.

The distinction between vaptan-resistant and vaptan-sensitive mutations is important for clinical therapy^3^ and emphasizes the value of molecular diagnostics in NDI and NSIAD management. However, it is still unclear how the diverse molecular defects identified in the two groups of NSIAD mutants contribute to the overall pathogenic effect of the mutated protein.

Given the rarity of NSIAD cases^16^, it is nearly impossible to establish meaningful correlations between type of mutation and severity of the disease, based on clinical data. Moreover, biochemical phenotypes observed in transfected cells fail to provide a rational scheme for predicting the disease-causing potential of the two groups of mutant receptors. Most intriguing in this regard, is the presence of a constitutive interaction with β-arrestin 2, which is a distinctive feature of vaptan-resistant mutations, but invariably lacks in all vaptan-sensitive mutations known to date. Arrestins desensitize GPCRs by blocking receptor-G protein interactions and accelerating receptor internalization^17,18^. Therefore, the constitutive interaction with β-arrestin 2 should mitigate the consequences of constitutive cAMP receptor signaling.

However, β-arrestins also act as independent signal transduction proteins that link GPCR signals to intracellular MAPK scaffolding^17–19^. Thus, the unrestrained activation of β-arrestin-mediated signaling may also contribute to the pathogenic effect of such mutations. If so, it is important to clarify whether constitutive activation of the receptor may involve only one or both β-arrestin isoforms, as the two proteins differ in the ability to enter the cell nucleus^20^ and may mediate divergent signaling pathways. However, no comparison of the interaction of V2R mutants with the two β-arrestin isoforms has been done to date.

A recent study in a mouse-derived renal cell line shows that both the extent of aquaporin 2 (AQP2) translocation to apical membrane and the enhancement of water permeability are well correlated with the increase of basal cAMP levels caused by a series of NSIAD mutants^21^. However, only F229V but not R137L/C mutations produced increased phosphorylation of S256 in AQP2, suggesting that codon 137 mutants might promote AQP2 translocation by a different mechanism than PKA-mediated phosphorylation^22^.

The interactions of a GPCR with different transduction proteins are often mutually exclusive, resulting in functional antagonism among diverse β-arrestin isoforms and Gs. This makes difficult to evaluate the effect of a mutation on constitutive and ligand-regulated interactions at each individual receptor-transducer interaction. In this study, we took advantage of cell lines derived from mice carrying deletions of β-arrestins or Gαs genes. By reintroducing fluorescent versions of single β-arrestin isoforms in mouse embryonic fibroblast (MEF) lacking both β-arrestin genes^23^, we compared the constitutive and agonist-regulated interactions of vaptan-sensitive and vaptan-resistant NSIAD mutations. We also assessed the role of each β-arrestin in promoting constitutive receptor internalization. Using Gs null fibroblasts^24^, we compared receptor endocytosis without Gs interference and verified the Gs-dependence of constitutive cAMP signaling in the mutations. Finally, we asked whether constitutive cAMP responses might depend on constitutive β-arrestins interactions by examining cAMP signaling in β-arrestin 1/2 null fibroblasts.

Here we show that R137 mutants exhibit strong preference of constitutive coupling to β-arrestin 2, despite the lack of β-arrestin selectivity for the agonist-driven interactions in the same receptors. We also show that constitutive cAMP signaling in R137 mutants does not depend on β-arrestins. Our data indicate that vaptan-sensitive and vaptan-resistant NSIAD mutations outline two different mechanisms of constitutive activation in GPCRs.

## Results

### Different role of β-arrestin 1 and 2 in mediating the constitutive internalization of V2 receptor mutants

For visualizing the effect of β-arrestin 1 (βarr1) and β-arrestin 2 (βarr2) on receptor trafficking, each MEF line expressing individual rGFP-tagged β-arrestin isoforms (see Methods) and the original β-arrestin1/2 KO line, were further transduced with R137H, R137L, F229V or wild-type V2R constructs, all fused to Dendra2. This green fluorescent protein can be irreversibly converted into a stable red fluorescent state upon irradiation with 405 nm light^25^, which allows resolving receptors from β-arrestins and assessing their intracellular co-localization in confocal imaging.

**Figure 1:**
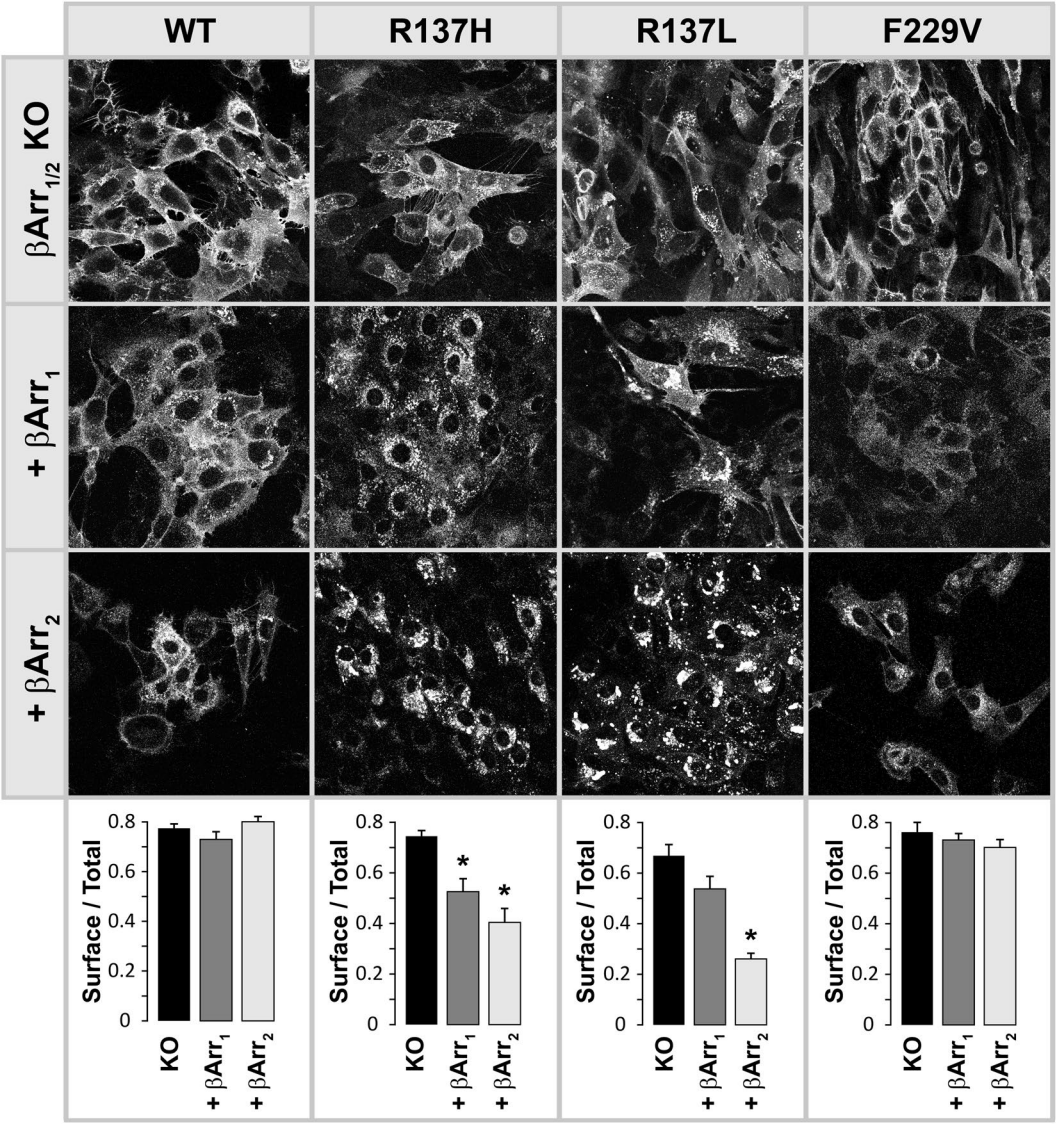
Cellular distribution of dendra2 fused wild type and mutant V2-receptors. Images are confocal sections of representative cell groups. Pictures in the 1^st^ row, representing β-arrestin 1/2 knock-out cells, are obtained from the green fluorescence signal of the dendra2 protein. In the presence of rGFP-tagged β-arrestin 1 (2^nd^ row) or β-arrestin 2 (3^d^ row), images are constructed using the red fluorescence signal of the photo-converted dendra protein to eliminate rGFP-tagged β-arrestin signals. See Supplementary Fig. S2 for separation of β-arrestin and receptor signals. Bottom: Quantification of surface/total expressions of the indicated receptor variants in the absence or presence of β-arrestin1/2 expression. These ratios were computed from the intracellular vs total fluorescence per cell, and averaged across the cells. Asterisks indicate statistical difference compared to the corresponding arrestin KO cells (Student’s t-test). One way ANOVA of the distribution ratios for WT and mutants in KO cells, yields no statistical significance. Note that in the absence of β-arrestin expression, cellular distribution of the receptors is similar in WT and mutants. The presence of β-arrestins minimally affects the basal distributions of WT and F229V mutant, whereas it clearly changes those of the R137H/L mutants (see text). The brightness was adjusted independently in each image, to improve visual perception of receptor distributions. Thus, fluorescence intensity across different images does not reflect the level of receptor expression.

In cells without β-arrestins, wild type (WT) and mutant V2Rs were mostly expressed on the cell surface (Fig. 1, top row). Although cell membrane contours are not well identified in these pictures due to the flat geometry of coverslip-attached MEF cells, we verified the preponderant membrane localization of WT and all mutants in freshly prepared round KO cells (Supplemental Fig. S1).

In βarr1 expressing lines, we found a more frequent occurrence of cells displaying intracellular receptors in small vesicles located in the perinuclear region (Fig. 1, mid row). Although such a pattern was more evident in Codon 137 mutations than WT or F229V, the expression of βarr1 did not cause a large difference in cellular distribution between mutants and WT, nor did it induce massive receptor clearance from plasma membranes.

In contrast, a sharp difference between vaptan-resistant R137L/H mutations and the vaptan-sensitive F229V mutation or WT receptor was evident in cells expressing βarr2 (Fig. 1, bottom row). While the cellular distribution of WT and F229V in βarr2 cells did not differ significantly from that observed in βarr1 cells, R137 mutants were not detectable on the cell surface, but only in intracellular bodies considerably larger than the perinuclear vesicles observed in βarr1 cells. The intracellular distributions of the NDI mutant R137H and the NSIAD mutant R137 L did not differ. Upon red conversion of the Dendra2 tag, there was full overlap of R137 mutants and βarr2 fluorescence in the vacuolar bodies (Supplemental Fig. S2), indicating that receptors and βarr2 are in close proximity in these structures.

**Figure 2:**
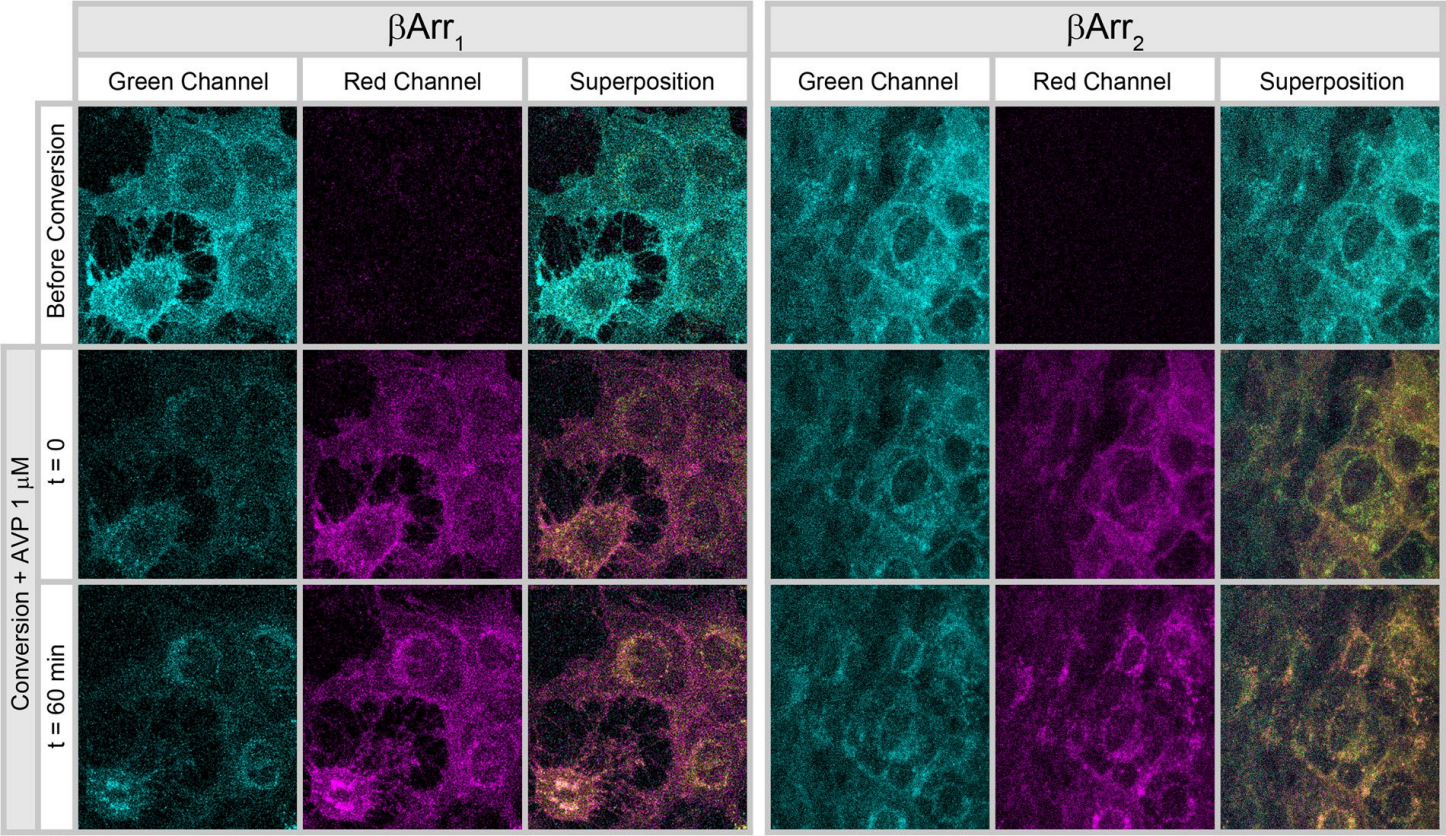
AVP-induced cellular redistribution of wild type V2R in cells expressing rGFP-tagged β-arrestin1 or β-arrestin2. Representative pictures are shown before (t = 0) and 60 min after application of 1 μM Arg-vasopressin (t = 60). Before dendra2 photo-conversion, the fluorescence signal in the green channel represents both receptor and arrestin proteins (dendra2 + rGFP). After photo-conversion (t = 0), green channel fluorescence mostly represents the distribution of β-arrestins (rGFP), whereas pure receptor signals (dendra2) appear in the red channels. Co-localizations of receptor and β-arrestins are seen as yellow signals in the superposed images. Experiments were carried out in KRH buffer at 37 °C. Note the formation of a punctuated perinuclear localization pattern of the receptor following the application of AVP (1μM). The apparent difference in fluorescence intensity between β-arrestin 1 and 2 in the picture (green channels after photo-conversion) does not reflect the difference in expression of the two isoforms, as the brightness of the pictures was adjusted independently in the two sets of cells. A microscopic quantification of β-arrestin 1 or 2 expressions in these cells lines is shown in Supplementary Fig. S3. Also, an approximate quantification of AVP-induced receptor internalization and β-arrestin colocalization is given in Supplementary Fig. S4.

To compare these large vacuolar structures with the physiologically formed vesicles of receptor endocytosis, we analyzed agonist-induced internalization of WT receptor in the presence of individual β-arrestin isoforms. AVP incubation (1μM, 1 hr) induced a punctuate pattern of receptor distribution into small perinuclear vesicles, in which β-arrestin and receptor were co localized (Fig. 2). There was no visible difference between βarr1 and βarr2 in that respect, and in neither case formation of larger intracellular clusters as observed when Arg 137 mutants are expressed in the presence of βarr2 was detected.

### Constitutive and agonist-induced coupling of V2R mutants to β-arrestins

**Figure 3:**
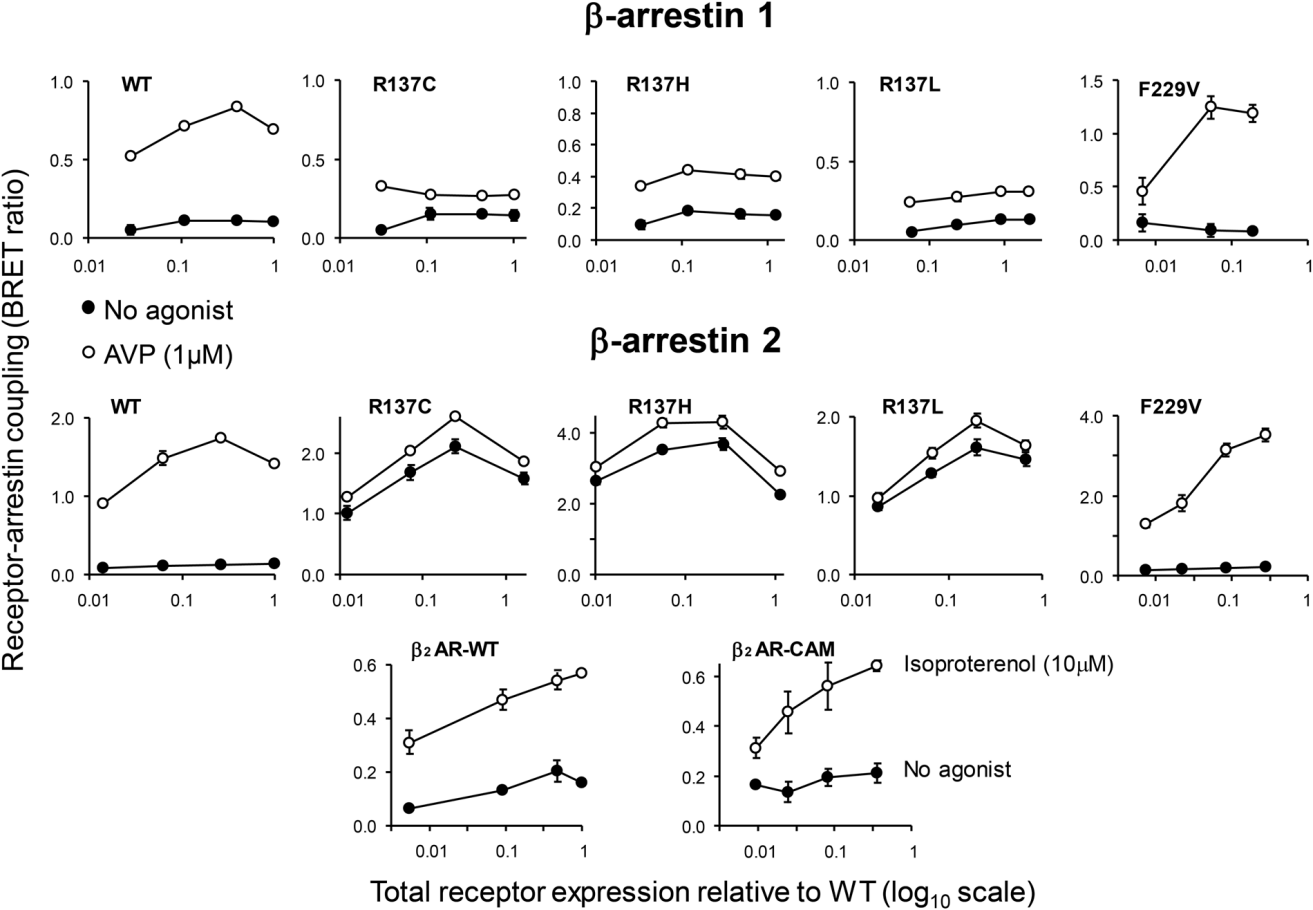
Receptor-arrestin interaction at increasing receptor expression. Retrovirally transduced β-arrestin 1/2 KO cells expressing rGFP-tagged βarr1 (top plots) or βarr2 (middle and bottom plots) were transfected with increasing concentrations of cDNA’s (0.8, 4, 20 and 100 ng/cm^2^) coding for Rluc-fused wild-type and the mutant V2Rs as indicated in each plot. For comparison, βarr2 expressing cells were also transfected with wild-type and costitutively active β_2_-adrenergic receptors (bottom plots). After 48 h of expression, the extent of receptor-arrestin coupling was measured in the absence and presence of agonist by BRET assay. The corresponding level of total receptor expressed was assessed by determining Rluc luminescence in the cell homogenates as described in Methods. In each row, levels of expressed mutant receptors are normalized to expression measured for wild-type receptor at the highest concentration of input cDNA. The data are means from 2–4 experiments each based on triplicate microwell transfections.

To verify the results of confocal analysis, we compared the strength of interaction between V_2_R mutants and individual β-arrestin isoforms using a BRET assay. We first studied the relationship between extent of receptor expression and arrestin-receptor coupling in the absence or presence of agonist. MEF lines expressing individual fluorescent β-arrestin forms were transiently transfected with increasing concentrations of vectors encoding rLuc-fused WT or mutant V2Rs. BRET ratios versus receptor expression (determined as total rLuc luminescence) are shown in Fig. 3.

All R137 mutants displayed a very high level of constitutive coupling to βarr2 but not to βarr1. The extent of constitutive coupling in R137L/C was comparable to the level of agonist-induced stimulation in WT V2R, and was even greater in the R137H mutant. Despite the enhanced constitutive interactions, all R137 mutants showed reduced sensitivity to AVP stimulation in both βarr1 and βarr2 cells. Neither WT nor F229V mutant receptors exhibited significant level of agonist-independent coupling for the two β-arrestins (Fig. 3). As a reference, we also studied βarr2 interactions of transiently transfected wild type and a constitutively active β_2_-adrenergic receptor mutant (β_2_CAM). This comparison can only be made in βarr2 expressing cells, as the interaction between β_2_AR and βarr1 generates no detectable BRET signal (Fig. 3, bottom). We found no difference in constitutive βarr2 coupling between β_2_CAM and WT β_2_-AR, indicating that the biochemical behavior of this prototypical activating mutation is more similar to F229V than to R137 V2R substitutions.

**Figure 4:**
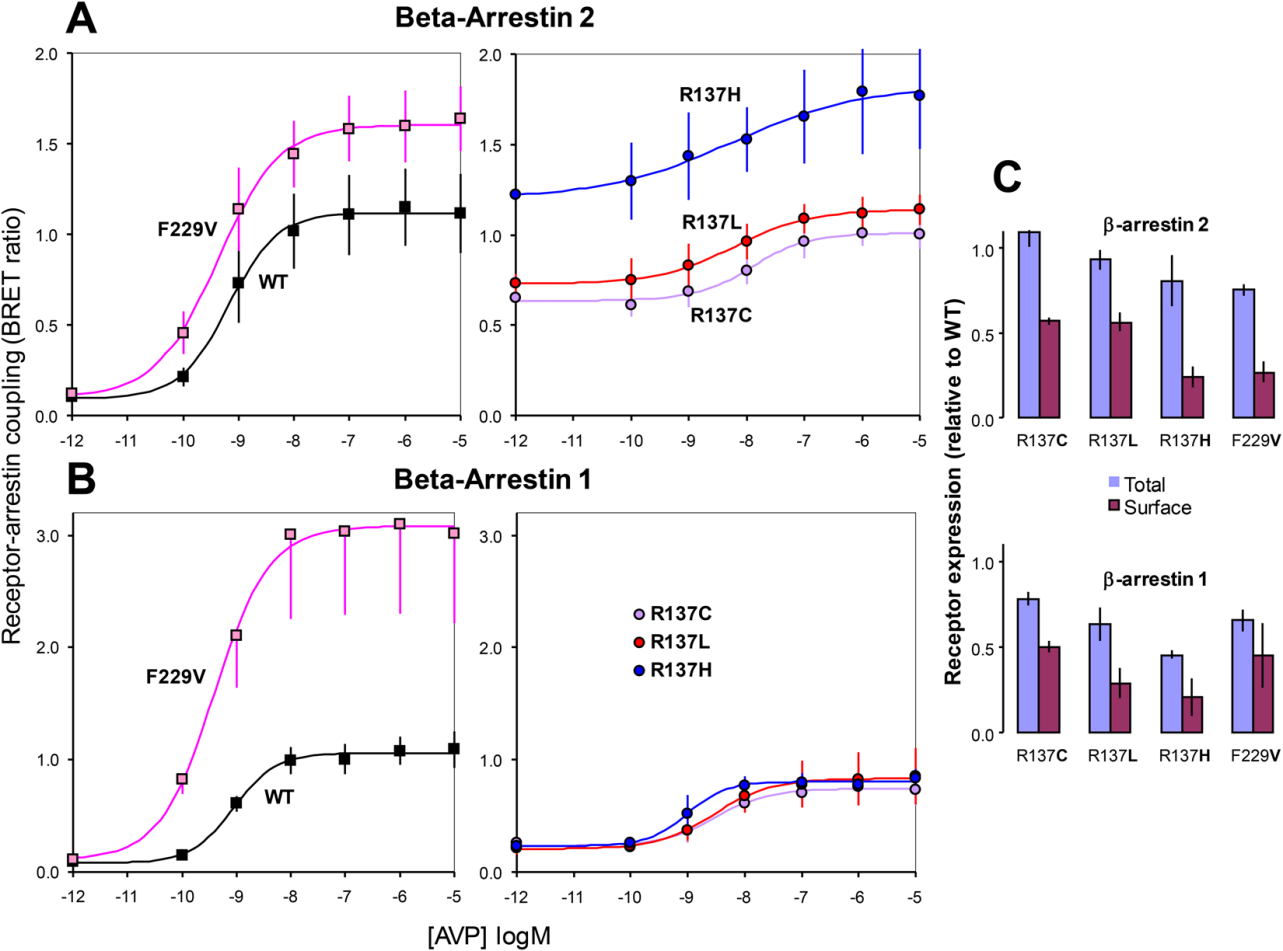
Concentration-response curves for AVP stimulated receptor-arrestin coupling. (**A**,**B**) Each curve represents the increase of BRET ratio as a function of AVP concentration (as log M) recorded in cells stably co-expressing rGRP-tagged β-arrestin 2 (**A**) or β-arrestin 1 (**B**) and rLuc-fused wild-type (WT) or mutated V2 receptors (as indicated in each plot). The curves of WT and F229V mutant are shown in separate graphs from R137 mutants, to improve clarity. Data points are means (±S.E.M) from 5 (WT, R137L, F229V) and 3 (R137C, R137H) experiments. The solid lines are predicted from fits with a 4-parameter logistic model (for parameters estimated see supplemental table 1). **C**: Means (±S.E.M) of total and cell surface receptor expression measured in the cells used in the experiments shown in A and B, determined using Rluc-tag luminescence and on cell ELISA, respectively, as described in Methods.

To further characterize the interaction, we examined agonist concentration-response curves in stable cell lines co-expressing each of the five luminescent V2 receptors with either βarr1 or βarr2 fluorescent proteins. All three R137 mutations, but not the F229V mutant, displayed 6–12 fold larger levels of constitutive coupling to βarr2 than WT V2R (Fig. 4A and SI Table S1). However, the net stimulation induced by agonist was decreased in R137 mutations and increased in the F229V mutant compared to the WT receptor. Moreover, the EC_50_ values of AVP in R137 mutants were ~10 fold greater than in WT or F229V receptors, confirming the reduced sensitivity to agonist activation of Arg substitutions (Fig. 4A and SI Table S1).

In βarr1 cells, R137 mutations exhibited only a small detectable level (~2 fold) of constitutive interaction, but other distinctive features, such as the decrease in net agonist stimulation and the increase in AVP EC_50_ values, were comparable to those observed in βarr2 cells (Fig. 4B and SI Table S1). The enhanced AVP response of the F229V mutant was more prominent in βarr1 than in βarr2 cells, although the difference was not significant, due to greater experimental variability of the curves recorded in βarr2 cells.

The total and surface expression of mutated receptors measured in the cell lines used for such studies were comparable in βarr1 and βarr2 expressing cells (Fig. 4C), indicating that the diversity in constitutive activity of R137 mutants for the two arrestins cannot be attributed to differences in receptor expression.

The effect of increasing concentrations of the inverse agonist tolvaptan on constitutive receptor-arrestin interaction was studied in cells co-expressing rLuc-fused WT, R137L, F229V and βarr2 (Supplemental Fig. S5). Even at very high concentrations, tolvaptan did not alter the constitutive BRET signal caused by R137L expression, nor changed the basal BRET ratio in WT and F229V receptors, where no constitutive βarr2 interaction occurs. However, the AVP concentration-response curves of all mutants were rightward-shifted by 2 orders of magnitude in the presence of 1μM tolvaptan, in both βarr1 and βarr2 expressing cells (Supplemental Fig. S5). This means that Tolvaptan can still bind with high affinity to WT and both mutant V2Rs, thus acting as a powerful competitive antagonist, despite the loss of negative efficacy in reversing the constitutive interaction of R137L with βarr2.

### Kinetics of receptor-arrestin interaction

**Figure 5:**
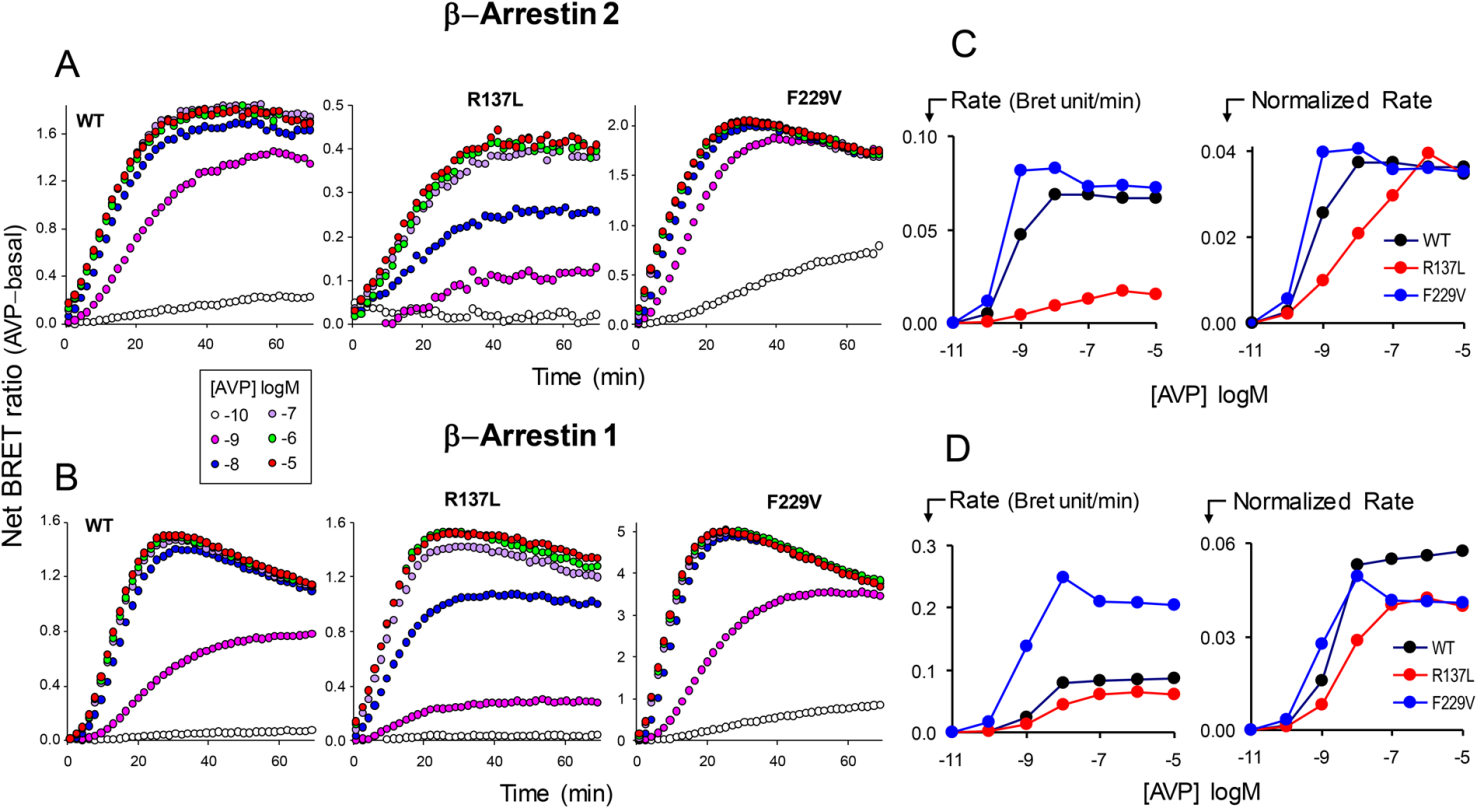
Kinetics of agonist-induced receptor-arrestin interactions in R137L and F229V mutants. A,B: Time course of agonist-induced receptor-arrestin coupling in cells co-expressing rGFP β-arrestin 2 (**A**) or β-arrestin 1 (**B**) and Rluc-fused wild-type (WT), R137L or F229V mutant V2 receptors. The increase of BRET luminescence following the addition of buffer or diffent AVP concentrations (as indicated in legend) was recorded for 70 min. The BRET ratio in the absence of agonist (basal) was subtracted from all data. **C, D:** Absolute rates of BRET increase at each ligand concentration in β-arrestin 2 (**C**) or β-arrestin 1 (**D**) cells calculated as slope of the initial linear part of the time course tracings are plotted as a function of AVP concentration (left plots). The right-most plots show the normalized rates obtained by dividing the absolute rates for the peak value of BRET enhancement measured in each receptor.

For investigating further the difference in agonist responsiveness between R137 and F229V mutations, we analyzed the kinetics of agonist-promoted receptor-arrestin association in cells co-expressing rLuc-fused WT, R137L and F229V receptors and βarr1 or βarr2. Figure 5(A,B) shows the time course of BRET signals in the presence of increasing AVP concentrations, while the initial rates of ligand-induced arrestin coupling computed from such time series are plotted as a function of agonist concentrations in Fig. 5C,D.

R137L and F229V mutations produced opposite changes in the intrinsic ability of agonist-bound V2 receptors to associate β-arrestins. The relation between rates of complex formation and AVP concentrations in R137L was shifted rightward compared to WT in both βarr1 and βarr2 cells, indicating an impaired ability of the agonist-receptor complex to bind arrestins. In contrast, the F229V mutant displayed equal rates of arrestin association at lower (βarr2) or similar (βarr1) AVP concentrations relative to WT. Thus, in the F229V mutation the intrinsic ability of agonist-bound receptor to associate arrestin is either enhanced or comparable to that of WT V2R.

### Constitutive and ligand-induced endocytosis of V_2_ receptor mutations

The Gs null fibroblast 2B2 cell line was engineered with a dual FYVE finger domain construct tethered to rGFP (see Methods) for measuring spontaneous and ligand-stimulated endocytosis of V2Rs. The 2xFYVE-GFP biosensor is primarily localized in early endosome vesicles^26^. Thus, changes in BRET ratio due to proximity between rLuc-tagged receptors and the fluorescent 2xFYVE biosensor can quantify the extent of receptor endocytosis without interference from Gs coupling.

Plasmids encoding rLuc-fused WT and mutants V2Rs were transiently transfected in the 2xFYVE expressing cells and BRET signals were recorded in the presence or absence of 1μM AVP (Supplemental Fig. S6). All R137 mutants displayed a significant increase of ligand-independent endocytosis and a reduction of AVP stimulation compared to WT receptor, whereas the constitutive and ligand-induced signal of the F229V mutant did not differ from WT.

These data confirm that R137 mutations but not F229V can increase the tendency of the V2 receptor to undergo constitutive endocytosis. However, the extent of increase in constitutive endocytosis was lower than the enhancement of constitutive interaction with βArr2 measured for such mutants.

### Effect of V_2_ receptor mutations on cAMP signaling

To test if V2R mutations may enable receptor control of cAMP signaling independent of Gs activation, we measured cAMP responses in Gαs KO cells expressing the luminescent cAMP biosensor Glosensor 22 F. Transiently transfected WT, R137L or F229V receptors did not alter cAMP accumulation (Supplemental Fig. S7) in absence or presence of AVP, indicating that neither mutant nor WT V2R can regulate intracellular cAMP in the absence of Gαs.

Next, we examined cAMP responses in the 2B2-Glosensor cells transduced with the large splice variant of Gαs, in order to reconstitute G protein-mediated control of adenylyl cyclase. High levels of the cAMP biosensor and Gs over expression are two sensitivity-enhancing factors that make this cell line particularly suited for appraising differences in constitutive cAMP signaling across V2 receptor mutants. In fact, both wild-type V2R and β_2_AR displayed detectable levels of inverse agonist-sensitive constitutive cAMP signaling in this cell line (Supplemental Fig. S8).

**Figure 6:**
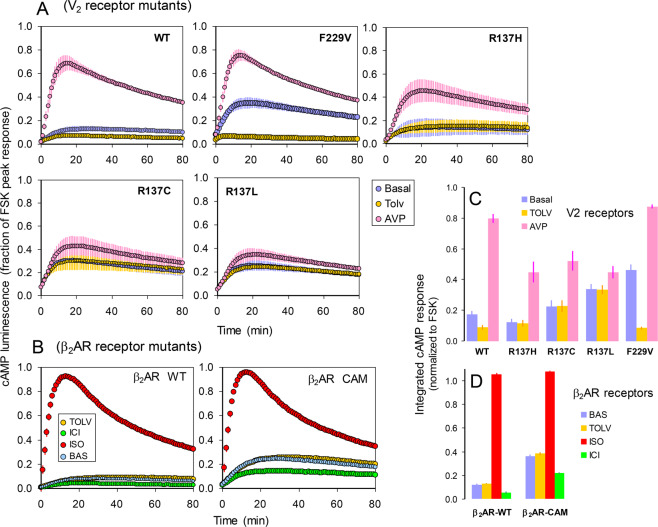
Constitutive and AVP-induced cAMP accumulation. 2B2 cells stably expressing Gαs and the Glosensor F22 were transiently transfected with cDNA’s encoding various V2 receptors (**A**) and β_2_ adrenoceptors (**B**) as indicated in each plot. The evolution with time of cAMP-induced luminescence was measured in the absence or presence of the agonists AVP (1μM) and isoproterenol (ISO, 10μM) or the inverse agonists, tolvaptan (tolv, 1μM) and ICI-118551 (ICI, 1μM), as indicated. The luminescence measured in mock-transfected cells was subtracted from all data and the resulting cps values were divided for the peak luminescence recorded with 100μM fosrskolin in the same transfected cells; data are means (± S.E.M) of three tracings recorded in a typical experiment. **C, D**: Integrated cAMP responses computed as areas under the curves from tracings obtained in cells transfected with V2Rs (**C**) or β_2_AR receptors (**D**). Bars are means (± S.E.M) of 9 luminescence tracings recorded in three independent experiments and were normalized to the response obtained with 100 μM forskolin in the same transfected cells.

After transient transfection with WT, F229V, R137H, R137C, and R137L mutants, cAMP luminescence was recorded in the presence of agonist (AVP, 1μM), inverse agonist (tolvaptan, 1μM) or forskolin (100μM) (Fig. 6A). The extent of cAMP response was quantified by time integration of the luminescence tracings (Fig. 6C). As a benchmark of constitutive activation, we also examined wild-type β_2_AR and the β_2_AR-CAM mutant in these experiments (Fig. 6B,D).

Compared to WT V2R, constitutive cAMP accumulation was markedly enhanced in F229V and to a lesser extent in R137L/C mutants, but not in the R137H mutant (Fig. 6A). However, all R137 mutations displayed diminished AVP stimulation and lack of basal tolvaptan inhibition, indicating reduced responsiveness to both agonist and inverse agonist. In contrast, AVP produced a response comparable to that of WT V2R in the F229V mutation, and tolvaptan strongly inhibited basal luminescence. Like F229V, the β_2_AR-Cam displayed enhanced constitutive cAMP accumulation and was responsive to both agonist (ISO) and inverse agonist (ICI-118551) (Fig. 6B,D).

**Figure 7:**
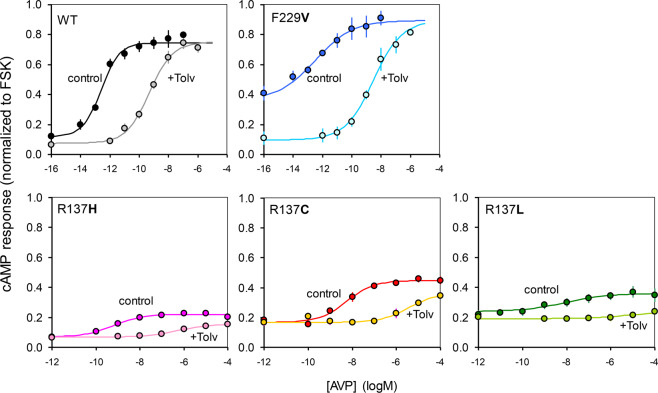
AVP Concentration-response curves for cAMP accumulation in presence and absence of inverse agonist. Integrated cAMP responses (computed as described in Fig. 7 in the same cells transiently transfected with V2 receptors), were measured at different concentrations of AVP in the absence and presence of 1μM tolvaptan. Data points are means (± S.E.M) of 5 (WT and R137L) or 3 (R137C,R137H and F229V) independent experiments.

Concentration-response curves of AVP confirmed the major loss of agonist efficacy in R137 mutations (Fig. 7). As shown by pEC_50_ values (SI Table S2), the picomolar potencies of AVP in WT and F229V V2R were reduced by nearly 3 orders of magnitude in R137 mutations, and the maximal effects of AVP were also diminished in such mutants (Fig. 7 and SI Table S2). However, tolvaptan was active as competitive AVP antagonist in all mutations (Fig. 7). Given the variability in EC_50_ estimates due to the narrow range of agonist response in R137 mutations, we could not quantify reliable pA_2_ values. Nonetheless, the presence of tolvaptan antagonism in all receptors indicate that no mutation can limit the access of inverse agonists to the V2R binding site, as also observed in β−arrestin cells (see Supplemental Fig. S5).

### cAMP regulation by V_2_ receptor mutants in the absence of β-arrestins

**Figure 8:**
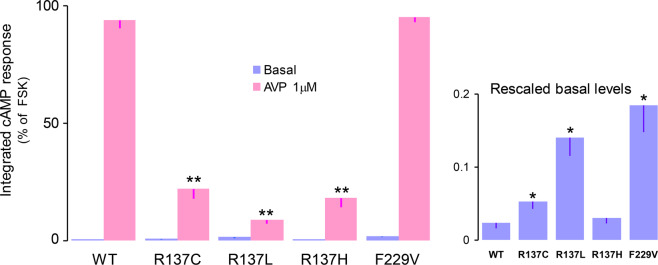
Constitutive and AVP-induced cAMP accumulation in the absence of β-arrestins. β-arrestin1/2 null MEF cells stably expressing Glosensor F22 were transiently transfected with cDNA’s encoding wild-type and mutants V2 receptors (as indicated) and cAMP signalling was measured in the absence and presence of AVP (1μM). cAMP responses were quantified as area under the curve of luminescence tracings recorded for 80 min. The corresponding integrated values measured in mock-transfected (which were significantly smaller than those in WT and R137H as determined by paired *t*-tests, P < 0.05) were subtracted. Data are expressed as % of forskolin response measured in the same cells. Bars are means (± S.E.M) of traces recorded in 3 experiments. The right side inset is a replot of the data measured in the absence of agonist. *P < 0.01 and **P < 0.001 for multiple comparison versus WT values as determined by one-way ANOVA followed by post-hoc Dunnet’s tests.

To evaluate if constitutive βarr2 coupling could mediate the constitutive cAMP activity of R137 mutations, we engineered a β-arrestin1/2 KO cell line permanently expressing the cAMP Glosensor. The effect of V2 mutations on cAMP signaling was determined by transiently expressing V2 receptors in this cell line. Due to the lower expression of endogenous Gαs and cAMP sensor, the visibility of constitutive cAMP effect in this MEF cell line was less prominent than in 2B2-Gs cells. Nonetheless, the pattern of cAMP signaling among V2 mutations was identical (Fig. 8). Ligand independent cAMP accumulation exceeded that of WT receptor in F229V, R137L and R137C, but not in R137H mutant. Conversely, AVP-mediated stimulation of cAMP was identical in F229V and WT receptors, but was strongly reduced in the three R137 mutations.

Thus, these data indicate that the constitutive effect of R137 mutations on cAMP signaling does not require the presence of β-arrestin isoforms in the cell.

## Discussion

Prior work^10–15^ has shown that vaptan-resistant R137 mutations, unlike the vaptan-responsive ones, result in agonist-independent interactions of V2R with βarr2. However, it was not clear if the same is true for βarr1 interactions. The only study that examined R137 mutants-βarr1 interactions reported that constitutive βarr1 recruitment depended on two different configurations of the donor/acceptor pair of the BRET assay used for detection. It was absent in one case but present in the other^14^. Although potentially interesting, this information certainly leaves the question unsettled. Moreover, no side-by-side comparison of the interactions with the two β-arrestins was ever made in that or subsequent papers.

Our results in cells expressing individual β-arrestin forms clearly show that there is an overwhelming difference between βarr2 and βarr1 in supporting constitutive interactions of R137 mutants. The spontaneous coupling of R137 mutants to βarr2 can approach or even surpass the extent of agonist stimulated WT V2R, while, in comparison, the constitutive interaction with βarr1 is negligible. Based on AVP concentration-response curves, we estimate that a small extent of constitutive coupling to βarr1 also exists in R137 mutants, but the level is only twice that of wild type V2R.

βarr2 is also far more effective than βarr1 in supporting the constitutive internalization of R137 mutants. Such mutants undergo massive sequestration into large intracellular clusters in βarr2-expressing cells, but only show a modest increase of constitutive endocytosis in βarr1 expressing cells. In agreement with previous reports^10–12^, neither wild type nor the vaptan-responsive F229V mutants display any constitutive interactions with the two β−arrestin isoforms. Altogether, our data indicate that all R137 substitutions impart to the V2 receptor a glaring ability to interact selectively with βarr2 in agonist unregulated manner.

In contrast, ligand-regulated coupling of R137 mutants to βarr2 and βarr1 are similar, and reveal a marked impairment of ligand control, as evidenced by the loss of inverse agonist inhibition of constitutive coupling and the reduced agonist stimulation, which was also the case for cAMP signaling. Conversely, the F229V mutation shows a noticeable increase of agonist responsiveness compared to WT V2R in both βarr2 and βarr1 interactions, although no agonist hyper reactivity is detectable in cAMP signaling.

The kinetics of agonist-driven receptor association to individual β-arrestin isoforms confirms the divergent changes of ligand regulation in the two groups of V2R mutations. The intrinsic rate of AVP-bound receptor association to both β-arrestins is reduced for R137L but increased for F229V, indicating that the two types of mutation can worsen and improve the efficiency of the receptor to form a ternary complex with arrestin.

The loss of ligand sensitivity in R137 mutants might be considered a result of constitutive internalization, since the drop in surface receptor density and the limited access of AVP inside the cell could both contribute in reducing agonist potency and maximal effect. However, the findings that the same loss of ligand sensitivity occurs in β-arrestins null cells where R137 mutants are in the plasma membrane and cannot undergo internalization exclude this possibility. This means that the decrease of ligand reactivity is a molecular property of the mutated receptors, not a consequence of altered intracellular trafficking.

In conclusion, our data suggest that the ligands ability to stabilize or destabilize the receptor-transducer complex (i.e., efficacy) is diminished in R137 mutations and enhanced in F229V. Thus, this fundamental difference in functional chemistry of the two NSIAD mutations groups is not limited to vaptan sensitivity, but underlies a global loss of ligand regulation, regardless of whether the ligand has positive or negative efficacy at the receptor. Based on these biochemical properties, the distinction between gain- or loss-of-function among mutations is also somewhat blurred, as all R137 substitutions result in similar loss of ligand efficacy, although exerting opposite pathogenic effects on renal function (i.e. NDI vs. NSIAD). However, it cannot be excluded that also R137H like the other R137 mutants can constitutively interact with Gαs. The fact that such constitutive interaction does not result in enhanced basal cAMP signaling might be explained by insufficient expression. In fact, as shown previously in a kidney cell line^21^, this receptor shows the lowest levels of both total and surface expression among R137 mutants.

The above considerations raise a number of questions on the existing relationship between observed biochemical behavior and the molecular defects caused by V2 receptor mutations.

One question concerns the mechanism of the selective constitutive interaction with βarr2 shown by R137 substitutions, despite the lack of β−arrestin selectivity when the interaction is driven by an agonist. How can a mutation impose β-arrestin discrimination in the absence, but not in the presence of bound agonist? One possible explanation may be the phosphorylation pattern of the receptor. There is evidence that distinct subsets of the seven GPCR kinases (GRKs) can generate diverse phosphorylation patterns, acting as “barcodes” that instruct β-arrestins to accomplish specific functions^27,28^. Accordingly, we may speculate that the pattern of constitutive phosphorylation in R137 mutations may promote selective engagement with βarr2 in the agonist-free receptor, but additional agonist-induced phosphorylations may restore unselective interactions with both β-arrestins. Although it is known that the R137H mutant is constitutively phosphorylated in the absence of ligand^29^, there is very limited information on whether different phosphorylation patterns can determine receptor discrimination between β-arrestin isoforms. Experiments based on mass fragmentation analysis of the phospho-peptides generated with and without agonist in R137 mutations, will be necessary to verify this hypothesis.

However, another possible explanation is that the large difference of constitutive BRET signals between βarr2 and βarr1 in R137 mutations may also reflect a diversity of the post-endocytic trafficking pathways where receptors are differently targeted by the two β-arrestins. For example, while βarr1 may direct the receptor to a recycling endosomal compartment where rapid separation from arrestin and membrane reinsertion occurs, βarr2 may drag the receptor to a vesicular compartment in which protein complexes accumulate and receptor-arrestin proximity may last much longer. This would generate a large difference of BRET signals at steady state. If so, R137 mutants might have low and similar levels of constitutive coupling to both β-arrestin isoforms, but the divergent fate of intracellular transit could dramatically amplify the difference in BRET ratios. The sharp contrast in intracellular trafficking patterns of R137 mutants between βarr1 and βarr2 shown in confocal imaging, and the disparity between extent of constitutive βarr2 coupling and early endosome translocation, are both in line with this interpretation. A recent study also suggests that the distributions of R137 mutants tend to overlap with typical markers of the non recycling endocytic pathways^12^.

The second question regards the constitutive cAMP signaling shown by R137C/L mutants. Could that result from constitutive β-arrestin interactions rather than from enhanced propensity to engage Gs in agonist-free state? This may seem a pointless question, as it is known that β-arrestins block GPCR coupling to G proteins^17,18^. In the resolved structure of a rhodopsin-arrestin fusion complex, the core interhelical socket where G proteins bind is occupied by the finger-loop domain of arrestin^30^, thus precluding any docking of G protein heterotrimers. In addition to this core, arrestins bind to the phosphorylated carboxyl terminal “tail” of receptors^31^, indicating that full stabilization of the GPCR-arrestin complex requires a two-step binding mechanism.

However, negative-stain electronmicroscopy studies have shown that GPCR-arrestin complexes can exist in two configurations, one with arrestin dually engaged to both tail and core sites, the other with arrestin only bound to the tail^32,33^. In the latter arrangement, the cytosolic pocket of the GPCR may still bind G proteins. There is indeed evidence that V2 receptors in partially engaged arrestin form can bind Gs and internalize as signaling competent “megaplexes”, where arrestin loaded with the dual receptor/Gs cargo can support G protein-mediated intracellular signaling^34^. Thus, we may suppose that constitutively phosphorylated R137L/C mutants can form Gs-bound megaplexes that generate intracellular activation of adenylyl cyclase and constitutive cAMP accumulation in the absence of agonist. In the R137H-arrestin complex, the balance between dual and single bound conformations might still favor the former, with negligible formation of Gs-bound megaplexes, which could explain why this receptor shows no substantial increase of constitutive cAMP signaling.

To test this hypothesis, we used β-arrestin1/2 null fibroblasts, as the lack of arrestin-mediated endocytosis should obviously preclude megaplex formation and thus abolish any constitutive cAMP formation of endocytic origin. The pattern of constitutive cAMP signaling in these cells is identical to that observed in cells expressing β-arrestins, indicating that the basal cAMP signaling of R137L/C mutants is not arrestin-mediated, but reflects the tendency of these receptors to interact constitutively with Gs. Thus, while the constitutive activation induced by F229V and other vaptan sensitive mutations is restricted to Gs, that induced by R137L/C mutations affects both Gs and β-arrestin interactions.

Finally, the last question refers to the mechanism generating two distinct constitutively active molecular phenotypes of the V2 receptor in NSIAD. The first type, including F229V and other vaptan-sensitive mutations, is characterized by greater level of constitutive activation, albeit confined to Gs interaction, and normal or enhanced responsiveness to agonists and inverse agonists. The second type, outlined by R137C and R137L mutants, is denoted by a major impairment of ligand reactivity and lower level of constitutive activation, which affects, however, a broader range of receptor-transducer interactions. As shown here, the pattern of constitutive and ligand-dependent signaling of the F229V mutant is similar to that exhibited by β_2_AR CAM, a constitutive active mutant that was extensively characterized in earlier studies^35^. This suggests that like β_2_AR-CAM, the first type of NSIAD constitutive activation may result from an increase in the equilibrium constants of the active states that are stabilized upon agonist binding.

However, in the second type of NSIAD constitutive activation a different mechanism is at play. Although R237C/L mutants are commonly defined as receptors “locked in the active state”, what seems to be really locked in such proteins is regulatory compliance to ligands. These receptors can no longer access the full conformational space of the macromolecule in response to ligand association, suggesting that the allosteric coupling between the ligand-binding pocket and the cytosolic region of the receptor is severely damaged. This leaves the receptor in a sort of weakly active state that does not appear to be matching agonist-induced conformations, as suggested by the concurrence of increased phosphorylation-dependent β-arrestin binding and the enhanced tendency to linger in a Gs-activating form.

Interestingly, type 1 constitutive activation is produced by substitutions of residues spread across several transmembrane receptor domains, which is consistent with the broad network of intramolecular interactions that support the allosteric change driven by the ligand. Instead, type 2 constitutive activation is only caused by substitutions within the E/DRY/H consensus sequence. Although codon 137 substitutions of NSIAD only target the basic Arg residue of this sequence, prior work in α_1_-adrenoceptors shows that site-directed replacements of D or R residues of the DRY sequence can both result in mutant receptors exhibiting increased constitutive activity with impaired agonist response^36,37^. Thus, the dual role of this consensus motif in both suppressing autonomous receptor activity and yet unleashing full control of receptor conformational changes to ligands, still needs further work to be completely understood.

In conclusion, our evidence based on knockout cells provides a clearer understanding of the relative contributions of altered receptor reactivity and cellular trafficking to the signaling properties of NSIAD mutations. We show here that constitutive arrestin recruiting and spontaneous Gs coupling are independent molecular features of R137 mutants that are detectable regardless of whether arrestins and Gs are co-expressed in the same cell. This excludes the possibility that constitutive cAMP signaling may be subordinate to spontaneous endocytosis. We also show that the faulty responsiveness of R137 mutants to agonists and inverse agonists is a direct result of mutation-induced changes on receptor reactivity not a consequence of intracellular receptor sequestration.

## Methods

### Reagents and cell lines

Cell culture media, reagents, and foetal bovine serum (FBS) were from Euroclone. Restriction enzymes from New England Biolabs. Coelenterazine and luciferin (Na salt) from Biotium Inc. or Nanolight Technology; VisiGlo HRP Chemiluminescent Substrate from VWR; mouse monoclonal antibody conjugated to peroxidase (anti-cmyc-peroxidase) from Roche; [Arg8]Vasopressin, Tolvaptan, Forskolin and all other biochemicals were purchased from Sigma-Aldrich. Pertussis toxin was from List Biologicals or Sigma-Aldrich. All other reagents were from Sigma-Aldrich, Merck or Fisher Scientific. The clonal fibroblast line isolated from mouse embryos carrying the targeted ablation of both β-arrestin 1 and β-arrestin 2 (MEF β-arr 1/2 KO cells)^23^ was made available to us by Prof. Robert J. Lefwokitz (Duke University, Durham, NC, USA). The clonal 2B2 cell line derived from mice carrying the targeted ablation of Gnas exon 2^24^ was supplied by Dr M. Bastepe (Harvard Medical School, Boston, MA, U.S.A.).

### Viral vectors and plasmids

Retroviral vectors encoding rLuc-tagged human cMyc-V2 receptors (wild-type and mutants, gifts of Prof. Michel Bouvier), rGFP-fused β-arrestin-1 and β-arrestin-2, and the long form of the human Gα protein GαsL were prepared as described previously^21,38,39^. The luciferase-based intracellular cAMP probe GloSensor 22 F was purchased from Promega and subcloned into puromycin resistance retroviral expression vector pQCXIP (Clontech). A vector encoding the 2xFYVE finger (consisting of a tandem repeat of residues 147 ± 223 from mouse Hrs with the QGQGS linker separating the two FYVE domains)^26^ was a gift of Dr. Harald Stenmark (Institute for Cancer Research, Oslo, Norway). A 2xFYVE-rGFP biosensor vector was made by linking the C-terminal of rGFP without stop codon to the 2xFYVE sequence and the construct was subcloned into the retroviral expression vector pQIXH (Clontech). Wild type and R137H, R137L, F229V mutant V2 receptors were fused at the C-terminal to the sequence of the fluorescent protein Dendra2^25^ (kindly provided by Dr. Konstantin Lukyanov, Institute of Bioorganic Chemistry, Russian Academy of Sciences, Moscow, Russia) by replacing their stop codons with a sequence encoding a 12-mer linker peptide (GSGGGGSGGGGS) and cloned into the retroviral expression vector pQIXP (Clontech).

### Cell culture and transfections

MEF β-arr 1/2 KO cell lines were cultured in DMEM (Dulbecco’s modified Eagle’s medium), 2B2 cell lines in a 50% mixture of DMEM and F12, both containing 10% FBS, in a humidified atmosphere of 5% CO2 at 37 °C. Cell lines stably co-expressing tagged receptors and the transduction proteins or biosensor constructs used in this study were obtained by infecting cells sequentially with retroviruses encoding the different fusion proteins followed by by appropriate antibiotics selections.

We first transduced β-arrestin1/2 KO cells with retroviral Hygro-resistant vectors encoding rGFP-β-arrestin 1 or rGFP–β-arrestin 2 and selected two MEF lines expressing similar levels of each β-arrestin isoform, as determined by fluorescence intensity. These lines were further engineered to generate cells useful to investigate the individual contributions of β-arrestins to internalization and signaling of V2R mutations. For confocal experiments, 12 stable lines were obtained by transducing the cells expressing individual rGFP-tagged β-arrestins and the original KO cells with pQIXP packaged retrovirus encoding Dendra2-fused WT, R137H, R137L, F229V V2Rs, followed by selection with puromycin (1 μg/ml) and/or hygromicin B (100 μg/ml). For BRET studies of receptor-arrestin coupling, the two MEF lines were infected with Puro-resistant retroviral vectors encoding rLuc-fused WT, R137L, R137C, R137H, F229V V2Rs, and 10 stable lines were obtained by selection with hygromicin B (100 μg/ml) and puromycin (1 μg/ml).

To generate Gs KO cells stably expressing the 2xFYVE-rGFP biosensor or reconstituted with exogenous GαsL, 2B2 cells were infected with Hygro-resistant virus encoding the respective constructs followed by hygromicin B selection (100 μg/ml).

For cAMP experiments, both 2B2 cells and β-arr 1/2 KO cells were infected with Puro-resistant retrovirus encoding the luciferase-based probe GloSensor 22 F, followed by puromycin selection (5 μg/ml and 1 μg/ml, respectively). To select cell lines with optimal expression of the biosensor, virally transduced cells were plated at very low-density in 15 cm dishes and 50–100 individual clones from each cell line were isolated with silicon rings for further screening. The cell clones with the best response to forskolin were identified by luminescence recording in 96 well plates.

Transient transfections of receptor cDNA’s were performed using linear polyethyleneimine (MW 25,000 Da, Polysciences, Inc) as described in^40^. Cells were allowed to express the genes for 48 h before starting cAMP experiments.

### Confocal microscopy

For fluorescence imaging, cells were either grown on glass cover slips, or transferred onto cover slips right before the experiments. All images were obtained in living cells by means of a confocal microscope system (Leica TCS SP5) equipped with argon-ion and He-Ne lasers, an additional mercury lamp in a diverse optical path, a 63x water immersion objective with a numerical aperture of 1.2 (Leica, HCX PL APO). All experiments were carried out in KRH buffer: (in mM) NaCl (120), KCl (4.7), CaCl_2_ (2.2), Hepes (10), KH_2_PO_4_ (1.2), MgSO_4_ (2), at room temperature, or at 37 C, as indicated in the text. Photoconversion of the dendra2 protein from green to red form was achieved by 10 seconds irradiation of the cells using the full spectrum of the 100 W mercury arc lamp (Osram, HBO 103w/2 model) mounted on the standard fluorescence excitation path of the microscope with diaphgram set at 10% attenuation. Such conditions did not produce artifacual changes in the pattern of receptor distribution, as determined by comparing dendra2-receptors expressed in MEF cells prior and after photo-conversion. The green and red forms of the dendra2 protein were detected by using, respectively, the 488 nm line of argon ion laser (Ex.) with the 500–540 nm band of the monochromator (Em.), and the 543 nm line of He-Ne laser (Ex.) with the 560 nm high-pass region of the monochromator (Em.). Simultaneous detection of green and red spectra was achieved by fast switching between the two configurations at each scanning line. Under such conditions, the green channel is blind to the red, and the red channel to the green form of the dendra2 protein. Data were collected with a spatial resolution of 1024 pixel per line with 8 bit of intensity depth and with a pinhole aperture of 1–1.5 Airy unit.

### Determination of total and surface expressed receptors

Total receptor expression was determined from the intrisic luminescence of the rLuc-tagged proteins as described previously^21^. Expression was quantified by linear regression analysis of titration curves obtained by plotting coelenterazine luminescence vs increasing amounts of cell protein homogenates. Data corrected for the number of living cells, as determined by Cell Counting Kit-8 (CCK8, Biotool) in parallel experiments, were normalized with respect to the expression of V2R wild-type. Cell surface expressed receptor were quantified on cell monolayers fixed under non permeabilizing conditions by a cell based chemiluminesent ELISA as described previously^21^. In 2B2 cells, cMyc immunoreactivity was revealed with an anti-cMyc AP conjugate antibody (Sigma) using VisiGlo AP as chemiluminescent substrate. Due to high levels of endogenous phosphatase activity in β-arrestin1/2 null MEF cells, an anti-c-myc HRP conjugated antibody and the chemiluminescent substrate Visiglo HRP were used in transfected lines derived from MEF cells.

### BRET assays of receptor–β-arrestins interaction and receptor endocytosis

BRET assays of receptor-arrestin coupling were performed and analyzed essentially as described in previous studies^38,39,41^.

For experiments in which coupling was studied at varying levels of receptor expression, rGFP-βarr1 and rGFP-βarr2 expressing cells grown in T25 flasks were transiently transfected with increasing amounts of cDNA encoding the various receptors, with the total amount of cDNA kept constant (20 ng/cm^2^) using non coding plasmid as filler. After 48 h of expression, cells were seeded in 96-well white plates (2 × 10^4^ cells/well) and further incubated 24 h before BRET assay.

For kinetics, cells grown in 96-well plates were pretreated with 5μM coelenterazine for 10 min. Then, AVP at varying concentrations was rapidly added with a multichannel pipet and the plate immediately transferred in the luminometer where the wells were continuously counted for 70 min.

To monitor V2R endocytosis, 2B2 cells stably expressing 2xFYVE-rGFP were transiently transfected with cDNAs (0.02 μg/cm^2^) encoding wild type and mutants V2R-Rluc. After 48 h, the BRET assay was started by replacing the medium with 50 µl of PBS containing 1μM AVP. After 15 min at 25 °C, 50 µl of PBS containing coelenterazine (10 µM final) were added to each well and cells were further incubated at 25 °C for 15 min before counting.

### cAMP measurements

MEF β-arrestins 1/2 KO or 2B2 cells stably expressing GloSensor-22F probe were transiently transfected with V2 receptors in T25 flasks. After 24 h of DNA expression, cells were seeded into 96-well white plastic plates at a density of 2 ×10^4^ cells/well and grown for additional 24 h. For cAMP assay, the wells were washed once with PBS, and further incubated for 60 min in 50 μl PBS containing 25 mM glucose and 2 mM luciferin. Next, 50 μl PBS containing 10μM Rolipram with or without varying concentrations of AVP or 100μM forskolin were added to the wells, and the plates were transferred in a luminometer (Victor Light, PerkinElmer). The total luminescence in each well (counts per second) was recorded at 30 s intervals for 80 minutes with 0.5 s integration time. The resulting tracings representing the evolution of cAMP luminescence with time are shown as fraction of the peak response measured for the corresponding trace in the presence of forskolin. To quantify overall cAMP response from such tracings, the area under the curve was calculated by numerical integration and the data are normalized to the corresponding response measured for forskolin.

### Analysis of the agonist CR curves

The AVP concentration-response curves obtained in BRET arrestin coupling and cAMP assays were fitted with a 4-parameter logistic function in log-form:

$$response=Bas+\frac{({E}_{\max }-Bas)}{1+{10}^{n(\log E{C}_{50}-\log [AVP])}}$$

Where, *Bas* and *E*_max_, are the response at vanishing and maximal AVP concentrations, respectively, *n* is the slope factor, and *EC*_50_ is the concentration of AVP yielding half-maximal response. Best fitting parameters obtained by regression analysis of the curves obtained in each experiment were averaged and are presented as means (± SEM) in table form (Supplemental Information, tables S1 and S2). The numbers of data points and replicates used in the analyses are indicated case by case in the tables.

## Supplementary information

Supporting information.
