# Supplementary material for: Vasopressin receptor 2 mutations in the nephrogenic syndrome of inappropriate antidiuresis show different mechanisms of constitutive activation for G protein coupled receptors
Source: Sci Rep. 2020 Jun 4;10:9111. doi: 10.1038/s41598-020-65996-w (PMC7272623; doi:10.1038/s41598-020-65996-w)
Supplement: Supplementary file 1 — Supporting information. [file 41598_2020_65996_MOESM1_ESM.pdf]

SUPPLEMENTAL INFORMATION for:

Vasopressin receptor 2 mutations in the nephrogenic syndrome of inappropriate antidiuresis show different mechanisms of constitutive activation for G protein coupled receptors

Vanessa Vezzi [1*], Caterina Ambrosio [1], Maria Cristina Grò [1], Paola Molinari [1], Gökçe Süral [2], Tommaso Costa [1], H. Ongun Onaran [2] and Susanna Cotecchia [3*]

[1] Istituto Superiore di Sanitá, Department of Pharmacology, Rome, Italy.

[2] Ankara University, Faculty of Medicine, Department of Pharmacology, Molecular biology and Technology Development Unit, Sıhhiye, Ankara, Turkey.

[3] Department of Biosciences, Biotechnologies and Biopharmaceutics, University of Bari, 70125 Bari, Italy

Content:

Figure S1. Receptor distribution in β-arrestin1/2 KO cells..page 2

Figure S2. Cellular distribution of Dendra2-fused V2 receptors
in cells expressing rGFP-βarr1 and rGFP-βarr2..page 3

Figure S3. Permanent expression of GFP-tagged β-Arr 1 or β-Arr 2 in MEF-KO cells............. page 4

Figure S4. Time course of fluorescence distribution after addition of 1 μM AVP.......................page 5

Figure S5. BRET analysis of the effect of tolvaptan on receptor-β-arrestin coupling..................page 6

Figure S6. BRET analysis of endocytosis...page 7

Figure S7. Basal and AVP-stimulated cAMP accumulation in Gs KO cells................................page 8

Figure S8. Basal cAMP after transient transfection of wild-type V2R and β_2AR
in 2B2-Gs cells ....page 9

Tables S1 and S2..page 10

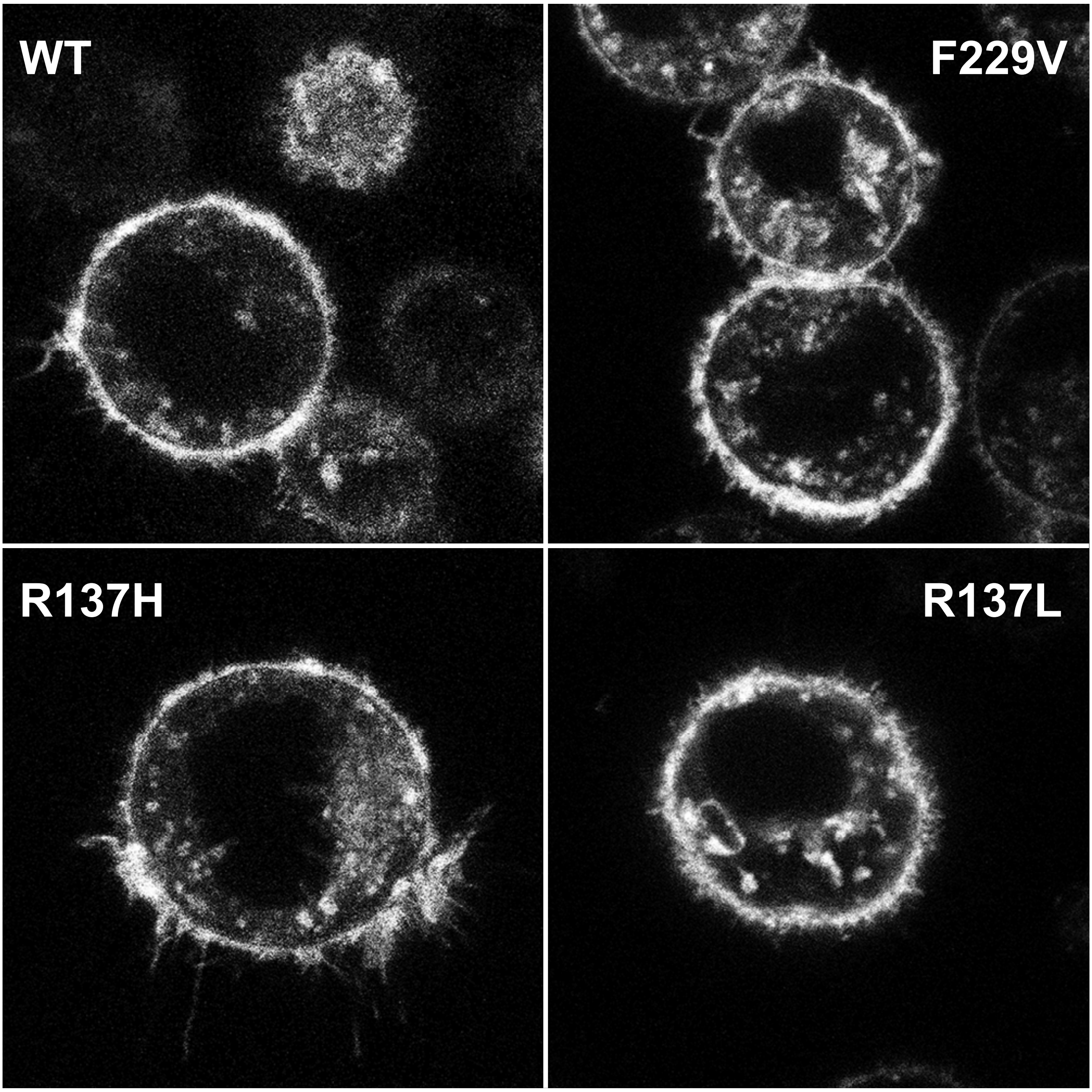

Figure S1 Receptor distribution in β-arrestin1/2 KO cells

Confocal images captured from β-arrestin1/2 KO MEF cells stably expressing Dendra2-fused V2Rs approximately 10 min after cell plating onto coverslips. Under such conditions the cells are firmly adherent to the glass but have not yet assumed the flat morphology that makes difficult to visualize the the plasma membrane in focal planes. Note the prevailing distribution of wild-type and mutant V2 receptors on the cell surface

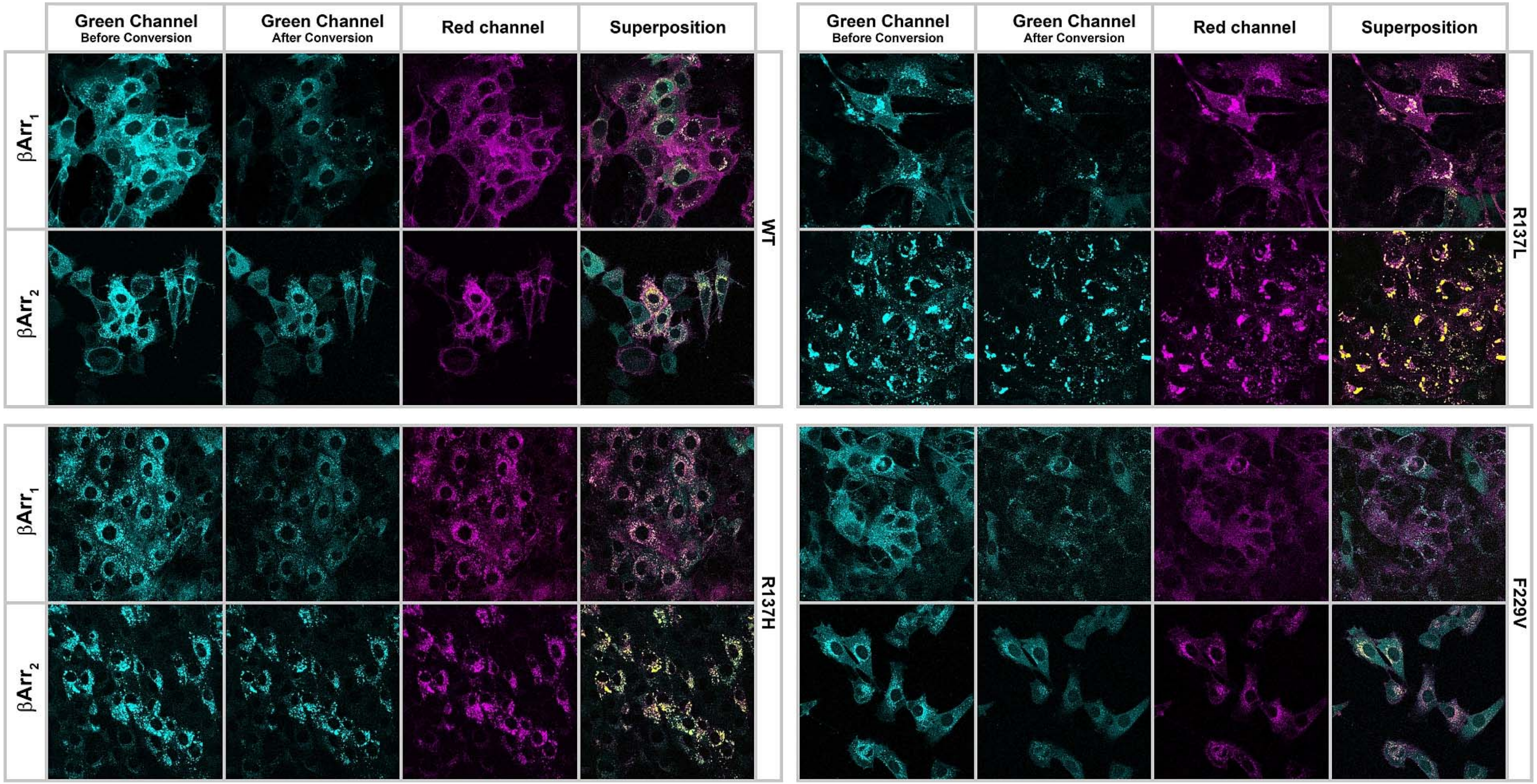

Figure S2 Cellular distribution of Dendra2-fused V2 receptors in cells expressing rGFP-βarr1 and rGFP-βarr2

The same data of Fig.1 in the main paper are shown after full decomposition of receptors and β-arrestins fluorescence. Each of the 4 panels corresponds to a different expressed receptor, as indicated on the right side. In the first column of each panel (labeled "Before Conversion") green fluorescence is the sum of signals from β-arrestins (rGFP) and receptors (dendra2, prior to red conversion). After red-conversion of Dendra2, the residual green fluorescence primarily reflecting β-arrestins-rGFP is shown in the second column of each panel, whereas the red-fluorescence emitted exclusively from receptors is shown in the third column. The fourth columns display the superposition of red Dendra2 and green rGFP signals, with yellow color indicating areas of receptors and β-arrestins colocalization. Note the difference in intracellular structures where R137 mutants and β-arrestins are co-segregated in βarr1 and βarr2 expressing cells.

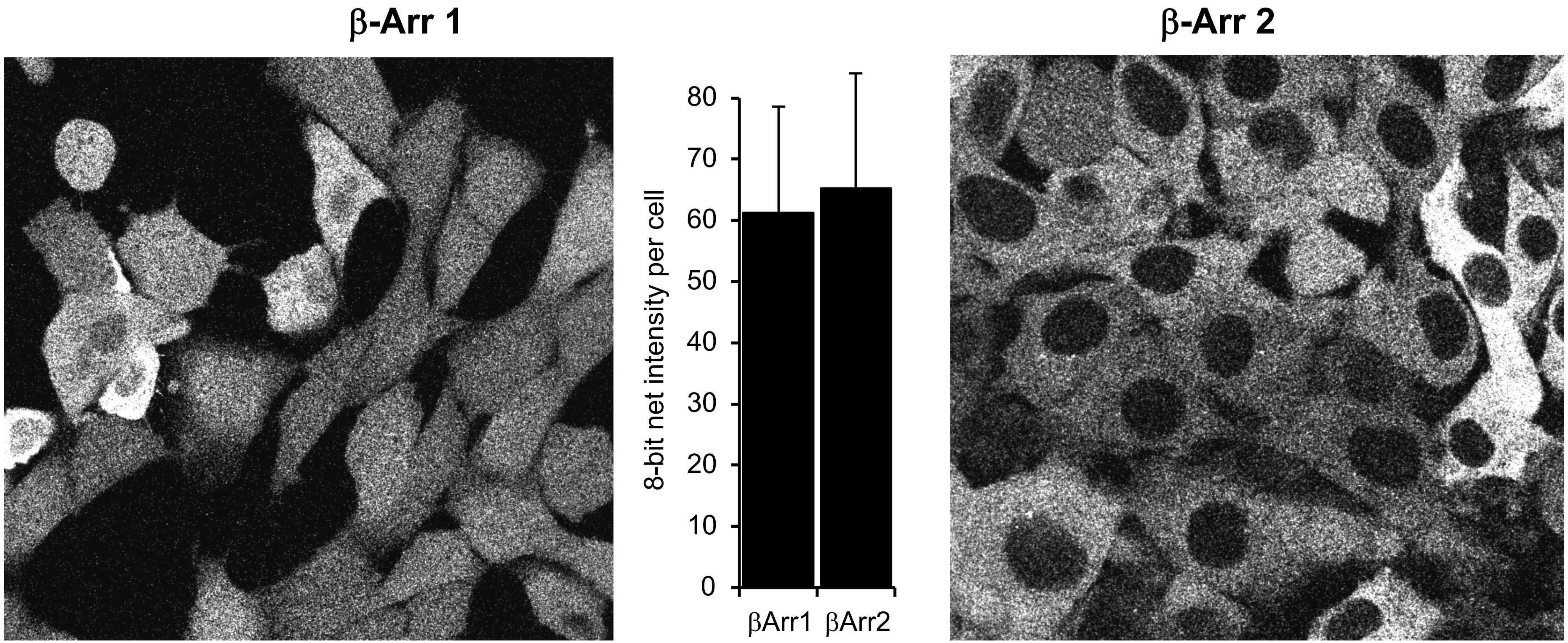

Figure S3 Permanent expression of GFP-tagged β-Arr 1 or β-Arr 2 in MEF-KO cells.

Confocal images of β-arrestin1/2 KO MEF cells that were virally transduced with either rGFP-βArr1 or rGFP-βArr2 are shown as indicated in the picture. Microscopic quantification of fluorescence of rGFP-tagged β-arrestin1 or 2 is given in the bar graph (middle). Aproximately 100 cells were evaluated for fluorescence intensities in the superposed confocal sections across the z-direction, and the results are given in terms of mean fluorescence per cell in 8-bit intensity units, as indicated in the picture. Variable expressions of β-arrestins among cells are evident in each case, but on average there is no significant difference between β-arrestin 1 and 2 expressions (as assessed by Student's t-test). Error bars in the graph showing variability among cells are standard deviations.

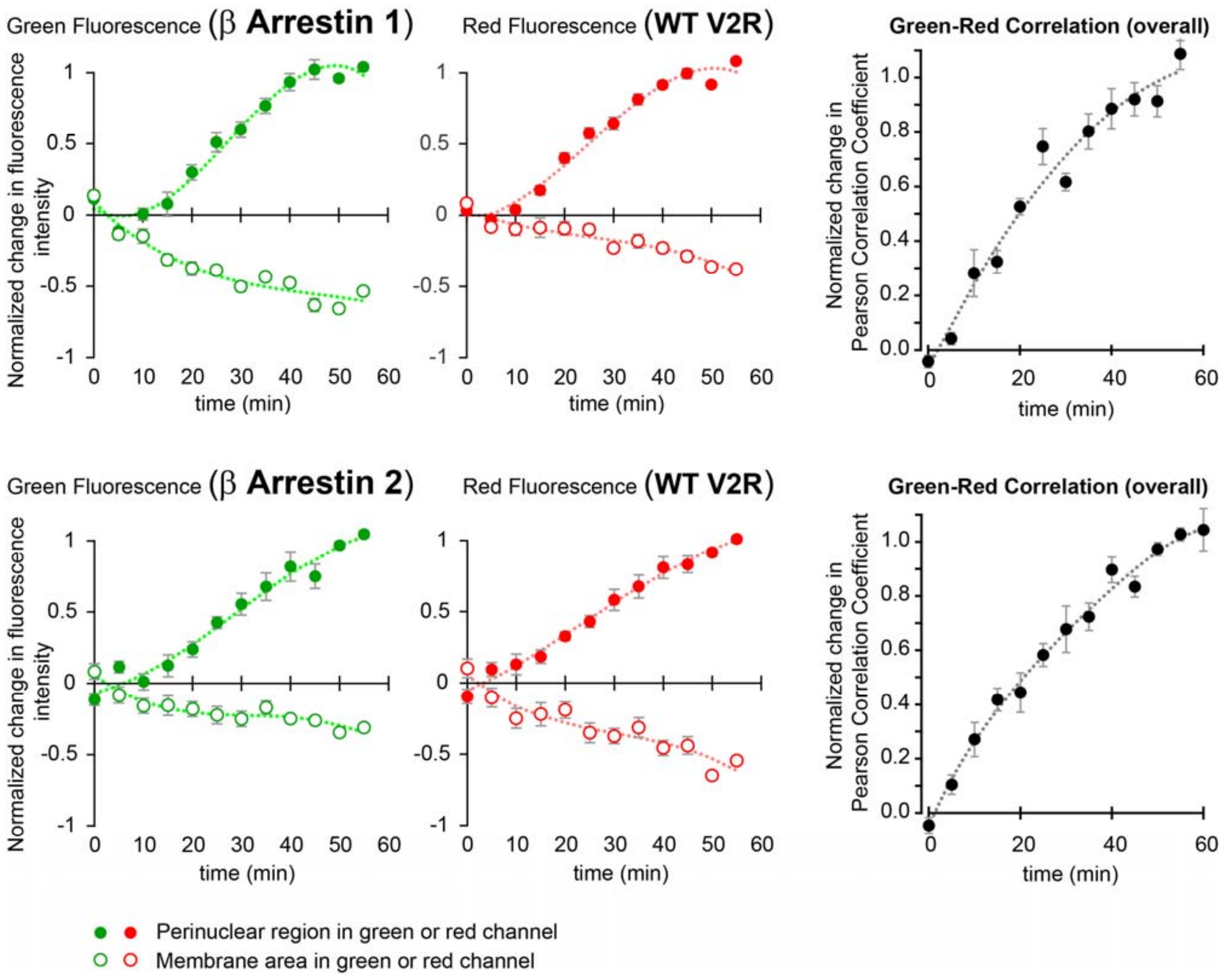

Figure S4 Time course of fluorescence distribution after addition of 1 μM AVP.

Time-dependent changes in cellular distribution of dendra-tagged WT V2R (red) and rGFP-tagged β-arrestin 1 or 2 (green) evaluated in MEF cells after addition (t=0) of 1 μM AVP (see Figure 2 in the main article).

The **first two columns** show AVP-induced, time-dependent changes in fluorescence intensities of WT-V2R and β-arrestins (βArr1 1st row, βArr2 2nd row). The recorded time-dependent fluorescence intensities from each cell in the region of interest (ROI) are normalized with respect to each initial (t=0) and final (t=60) value, and then averaged across all cells (n=12-17). The curves for membrane area (*open symbols*) are rescaled according to the averaged final density difference between membrane and corresponding perinuclear region. ROI's for each cell were visually selected from the images. ROI's designated as "membrane area" include cell membrane and part of the nearby cytoplasm away from the perinuclear region. ROI sizes were variable depending on the cell shape, but recorded intensities are normalized with respect to each ROI size (i.e. they are density values). Note that both in case of β-arrestin1 and 2, perinuclear densities of receptor (red) and β-arrestin (green) increase, while near-membrane densities decrease.

The **third column** shows time-dependent AVP-induced co-localization of receptor and β-arrestin (βArr1 1st row, βArr2 2nd row). To measure co-localization, we calculated at each time point pixel-wise Pearson correlation coefficients between red and green signals in whole cells (Dunn, K.W., et al. *Am. J. Physiol. Cell Physiol.* 300, C723–C742, 2011). For each cell, the correlation coefficients were normalized with respect to initial and final values, and then averaged across all cells to obtain the curves shown in the picture. Note that not only the density of receptor and β-arrestins in the perinuclear area, but also their overall co-localization increases upon addition of AVP. Error bars are S.E.M.

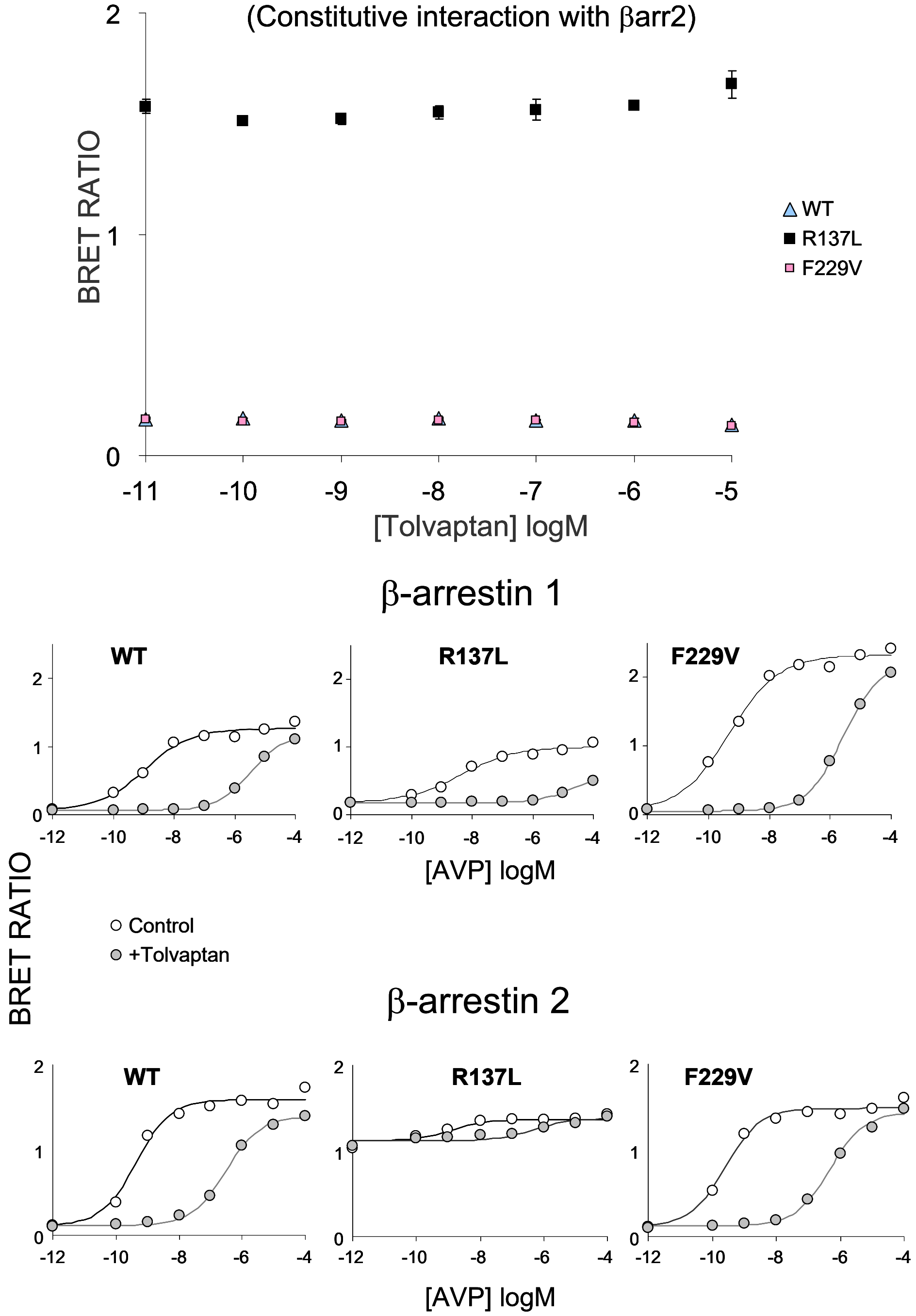

Figure S5 BRET analysis of the effect of tolvaptan on receptor-β-arrestin coupling

The top graph shows BRET ratio signals recorded under increasing concentrations of the inverse agonist tolvaptan in the absence of agonist, using cells co-expressing rGFP-βarr2 with rLuc-tagged wild-type (WT), R137L or F229V mutant receptors. The lower panels show AVP concentration-response curves for promoting receptor-β-arrestins interaction that were obtained in the absence and presence of tolvaptan (1μM). Cells co-expressing rLuc-tagged receptors (as indicated in each plot) with rGFP-βarr1 (top row) or rGFP-βarr2 (bottom row) are compared,

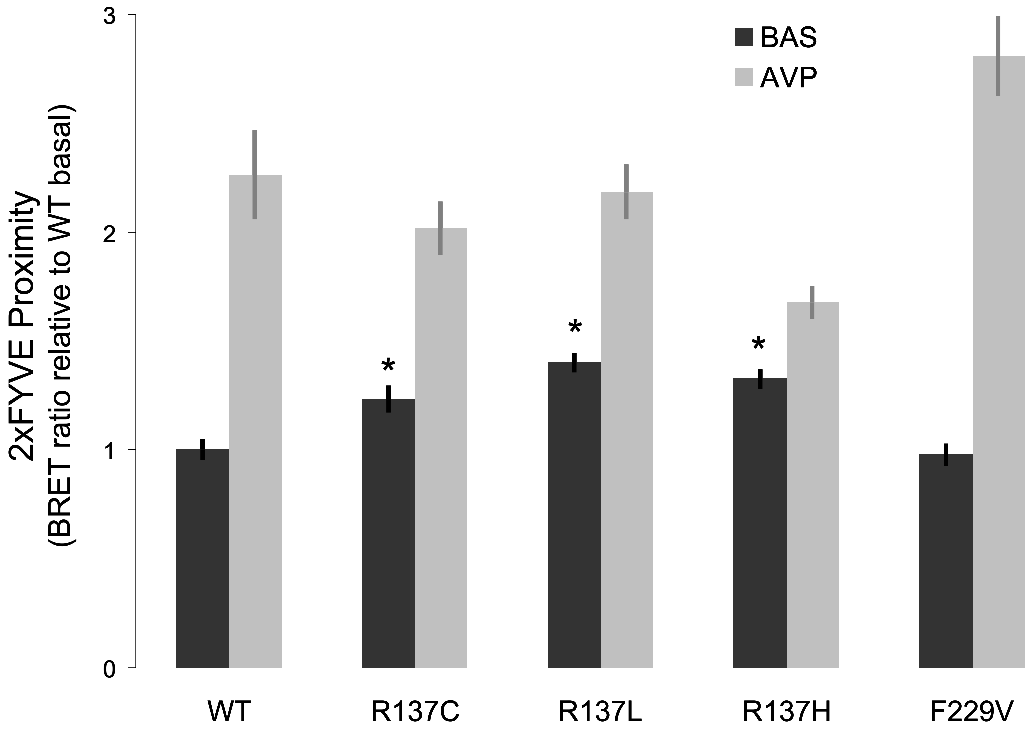

Figure S6 Endocytosis of V2 receptors in absence and presence of agonist.

2B2 cells stably expressing 2xFYVE-rGFP were transfected with plasmids encoding Rluc fused V2 receptors: WT, R137C, R137L, R137H, and F229V. After 48h of expression, the cells were incubated in the absence (BAS) or presence of agonist (AVP, 1μM, 15 min) and the BRET ratio indicating the extent of molecular proximity between the receptor and the 2xFYVE biosensor was determined (see Methods). All BRET ratios were divided for the value recorded in WT receptor without AVP. Data are means (± S.E.M.) of 4 independent experiments. * $P < 0.01$ versus WT basal, as determined by one-way ANOVA of the basal values and post-hoc analysis with Dunnet's test for multiple comparisons.

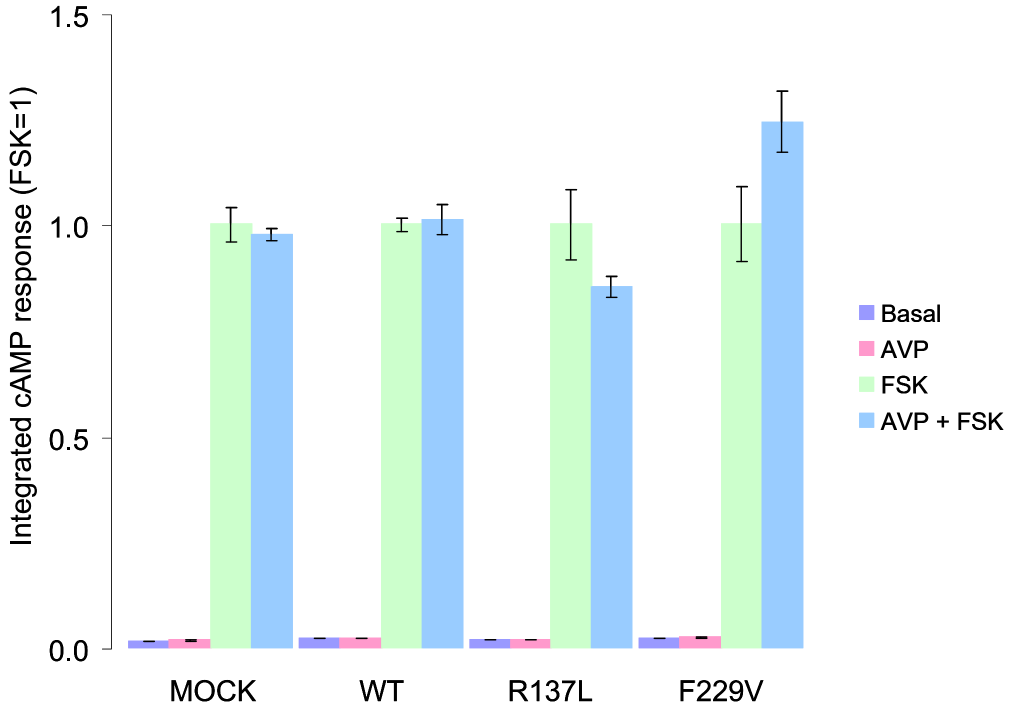

Figure S7 Basal and AVP-stimulated cAMP accumulation in Gs KO cells.

2B2 Gs KO cells stably expressing the cAMP probe Glosensor F22 were transiently transfected with cDNAs encoding V2 receptors (as indicated) or non-coding cDNA (MOCK). cAMP luminescence was recorded after 48h of expression (see Methods) in the absence (Basal) or presence of AVP (1μM), forskolin (FSK, 100μM) or both AVP and FSK. The cAMP responses were quantified as area under the curve of the luminescence cps tracings recorded over 80 min and are normalized to the response obtained with FSK in the mock-transfected cells. Data are means (± S.E.M) of triplicate determinations.

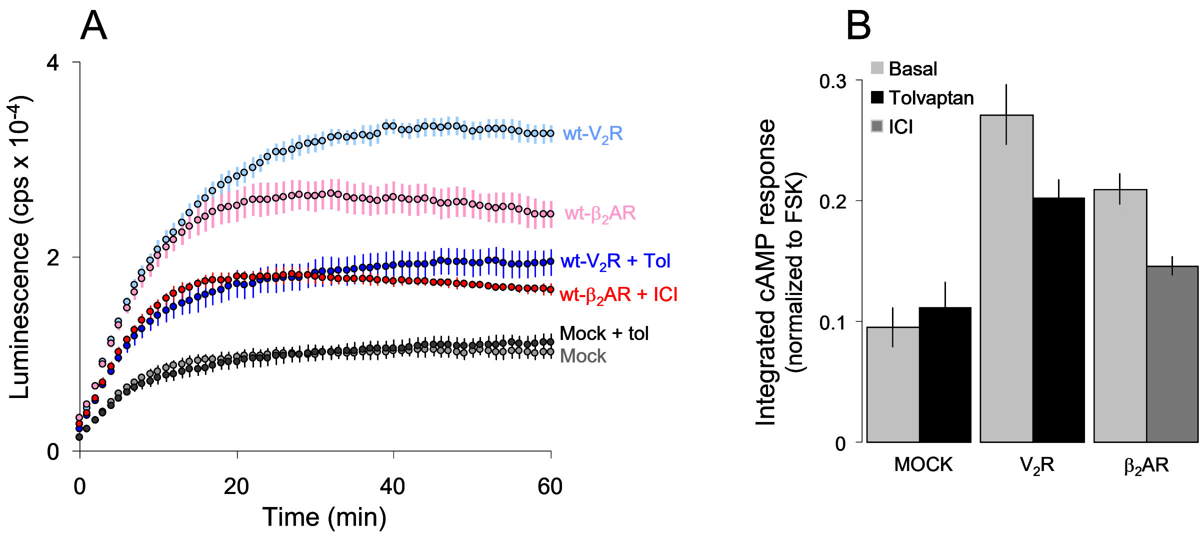

Figure S8 Basal cAMP after transient transfection of wild-type V2R and β_2AR in 2B2-Gs cells.

2B2 cells stably expressing the Glosensor F22 probe and the Gαs large variant were transfected with non coding cDNA (MOCK) or plasmids encoding wild-type V2R or β_2AR. **A** cAMP luminescence recorded in the absence or presence of the inverse agonists, tolvaptan (tol, 1µM) or ICI 118,551 (ICI, 1µM), and forskolin 100µM (not shown in the plot). **B** cAMP response quantified as area under the curve normalized to forskolin. Data are means (± S.E.M) of triplicate determinations.

SUPPLEMENTAL TABLES

Table S1: Best-fitting parameters computed from concentration-response curves of AVP for stimulating receptor-arrestin coupling (see Figure 4). Emax and Basal are in BRET ratio units. Values are means (± S.E.) derived from *n* curves as reported for each receptor

	**β-arrestin 2**				
	**WT** *(n=6)*	**F229V** *(n=5)*	**R137C** *(n=3)*	**R137L** *(n=5)*	**R137H** *(n=3)*
Emax	1.12 (0.21)	1.60 (0.18)	1.01 (0.08)	1.14 (0.09)	1.82 (0.29)
Basal	0.09 (0.02)	0.10 (0.02)	0.64 (0.05)	0.73 (0.08)	1.20 (0.22)
log(EC_{50})	-9.18 (0.12)	-9.39 (0.19)	-7.91 (0.15)	-8.21 (0.42)	-8.24 (0.54)
	**β-arrestin 1**				
	**WT** *(n=6)*	**F229V** *(n=4)*	**R137C** *(n=3)*	**R137L** *(n=4)*	**R137H** *(n=3)*
Emax	1.06 (0.14)	3.08 (0.78)	0.74 (0.07)	0.83 (0.07)	0.80 (0.08)
Basal	0.08 (0.01)	0.11 (0.04)	0.23 (0.02)	0.21 (0.02)	0.23 (0.03)
log(EC_{50})	-9.05 (0.08)	-9.40 (0.17)	-8.51 (0.08)	-8.52 (0.05)	-9.00 (0.06)

Table S2: Parameters computed by fitting concentration-response curves of AVP for stimulating cAMP accumulation in 2B2 cells (Figure 7). Basal and E_{max} are expressed as fraction of response measured in the presence of 100 μM forskolin in the same transfected cells. Data are the means (± S.E.) of the best fitting estimates obtained from 5 (WT) or 3 (mutants) concentration-response curves

	Control			+ Tolvaptan (1μM)			Δ pEC_{50} (Control – Tolv.)
Receptor	Basal	E_{max}	pEC_{50}	Basal	E_{max}	pEC_{50}	
WT	0.12 ± 0.02	0.74 ± 0.04	12.6 ± 0.16	0.07 ± 0.02	0.75 ± 0.04	9.30 ± 0.18	3.31 ± 0.24
F229V	0.36 ± 0.06	0.89 ± 0.07	12.5 ± 0.49	0.10 ± 0.03	0.89 ± 0.03	8.52 ± 0.13	3.98 ± 0.51
R137C	0.17 ± 0.04	0.45 ± 0.02	8.2 ± 0.33	0.17 ± 0.04	0.31 ± 0.02	5.88 ± 0.34	2.37 ± 0.48
R137L	0.23 ± 0.03	0.36 ± 0.03	8.0 ± 0.62	0.19 ± 0.01	0.23 ± 0.02	5.21 ± 0.52	2.82 ± 0.81
R137H	0.07 ± 0.03	0.22 ± 0.04	9.2 ± 0.45	0.07 ± 0.02	0.16 ± 0.03	6.24 ± 0.51	2.99 ± 0.68
